# The PBC Model: Supporting Positive Behaviours in Smart Environments

**DOI:** 10.3390/s22249626

**Published:** 2022-12-08

**Authors:** Oluwande Adewoyin, Janet Wesson, Dieter Vogts

**Affiliations:** Department of Computing Sciences, Nelson Mandela University, Port Elizabeth 6031, South Africa

**Keywords:** behavioural problems, smart environments, user modelling, behavioural modelling, personal smart environments, classification

## Abstract

Several behavioural problems exist in office environments, including resource use, sedentary behaviour, cognitive/multitasking, and social media. These behavioural problems have been solved through subjective or objective techniques. Within objective techniques, behavioural modelling in smart environments (SEs) can allow the adequate provision of services to users of SEs with inputs from user modelling. The effectiveness of current behavioural models relative to user-specific preferences is unclear. This study introduces a new approach to behavioural modelling in smart environments by illustrating how human behaviours can be effectively modelled from user models in SEs. To achieve this aim, a new behavioural model, the Positive Behaviour Change (PBC) Model, was developed and evaluated based on the guidelines from the Design Science Research Methodology. The PBC Model emphasises the importance of using user-specific information within the user model for behavioural modelling. The PBC model comprised the SE, the user model, the behaviour model, classification, and intervention components. The model was evaluated using a naturalistic-summative evaluation through experimentation using office workers. The study contributed to the knowledge base of behavioural modelling by providing a new dimension to behavioural modelling by incorporating the user model. The results from the experiment revealed that behavioural patterns could be extracted from user models, behaviours can be classified and quantified, and changes can be detected in behaviours, which will aid the proper identification of the intervention to provide for users with or without behavioural problems in smart environments.

## 1. Introduction

The introduction and implementation of the Internet of Things (IoT) have allowed connectedness between physical and virtual entities. IoT is a medium where daily devices become smarter, processing becomes more intelligent, and everyday communication becomes clearer [1]. IoT is a large-scale architecture for the information society, allowing state-of-the-art services by connecting virtual and physical things built on existing, developing and connectable information and communication technologies [2]. The IoT concept has been known for its impact in providing many solutions in several domains [1]. From Gartner’s report, more than 25 billion devices will be connected through the IoT by the end of 2021 [3], and there will be an increase in 2022 [4]. In the year 2020, with the outbreak of COVID-19, the Internet of Behaviour (IoB) emerged as office workers resumed work after the lockdown. IoT devices increased in office environments to monitor workers’ compliance with COVID-19 protocols [5]. IoT paved the way for providing solutions to several behavioural problems by utilising data to modify workers’ behaviours.

A systematic review of two studies revealed that 54% of adults in Slovakia have behavioural problems with healthy food consumption and physical activity [6]. Three studies in Slovakia showed that 38% of the total population has behavioural issues with vegetable consumption and smoking. The presence of alcohol misuse and smoking was identified in adults, with a prevalence ratio of 2.89. These statistics were significantly higher in the US, as 10% of young adults (18–25) identified behavioural problems, such as alcohol misuse, in 2017. Petersen [7] reported that 49% of young adults in South Africa were exposed to alcohol abuse before the age of 18, leading to behavioural problems in driving, eating, and violence. There is a need to monitor the behaviour of individuals to identify such issues because of their high incidence and lifelong effects on people, and these are often reflected in many domains, for example, health, resource usage, and office work productivity.

Behavioural modelling has been used to provide solutions to behavioural problems. Behavioural modelling refers to the observation of an individual in certain situations for good and poor behaviours, which generally involves behavioural data collection [8]. The description and manipulation of human behaviour are fundamental to many domains because they help to identify and understand good behaviours and to ensure their sustainability over time. They also identify those behaviours eliciting poor behavioural patterns in people and how these poor behavioural patterns can be modified through discovering, investigating, monitoring, and changing human behavioural patterns [9]. Behavioural modelling occurs in two aspects. The reinforcement of good behaviours, if good behavioural patterns are observed, through the development of sustainable behaviours over a long period, and the provision of adequate interventions for modification if poor behavioural patterns are observed. These two aspects justify behavioural modelling and can be applied to several domains, for example, the office domain, which is the focus of this study. 

In behavioural modelling using an SE, there are essential concepts that require proper understanding. These are activity, behaviour, good behaviour, and poor behaviour. Fatima [10] defined activity as an action or task performed by a user at a time. An SE is an environment equipped with smart devices such as sensors and sensor networks interconnected through the IoT to provide its users with assistance, maintenance, and services [11]. SEs can be implemented in any form, depending on the purpose for which they were designed. This can be a smart home, smart office, smart city, or a personal smart environment, a group of services accessible within an active space of linked devices, owned and administered by a single user. 

Guez [12] defined behaviour as the means by which individuals act, conduct, or express themselves. Pate [13] described behaviour as a reaction to an object that is directly or indirectly observable. Direct observation refers to studying an entity in a physical environment, while indirect observation refers to making decisions and describing a result. These definitions describe behaviour with respect to objects, people, and social norms or how an individual handles and relates to other individuals and entities within an environment. This study will adopt the definitions by [14,15]. Wallace [16] defined behaviour as the recognisable activities of an individual that can involve action and reaction to stimulation. Soto-Mendoza [17] defined behaviour as a recurring series of activities relative to the frequency of recurrence over an extended period.

Good behaviours are behaviours that are generally accepted by individuals or society. They refer to the acceptable conduct of an individual’s emotional, physical, and cognitive processes in a context or environment. A collection of good behaviour generates good behavioural patterns. Good behavioural patterns are equivalent to normal behaviour patterns as they refer to behaviours consistent with a person. The behaviours can vary for an individual, context, location, time, and societal norms [18]. Poor behavioural patterns are studied in contrast to good behaviour patterns and, in most cases, lead to behavioural problems. Behavioural problems in people have created many difficulties in health, home, and offices. However, this study will explore low productivity as a significant behavioural problem in offices that need behavioural modelling. Within office environments, multitasking/interruption and social media are the major factors that affect people’s work productivity. These are behavioural problems because many individuals simultaneously engage in many activities, leading to slow task progress. Workers tend to focus on one task but are interrupted by another task or a colleague. As Pataki-Bitto [19] explained, workplace interruptions have many effects and types. The results can be time loss, error, emotional drain, or mental workload.

These behavioural problems have been previously solved through traditional monitoring of individuals via self-reporting [20] and behavioural modelling using smart environments. This study will focus on the latter because of its capability to minimise biases common to the former. There are many studies on behavioural modelling using smart homes, and most of these studies are data-driven in nature. For example, Lazzari [21] modelled energy consumption behaviours through a smart meter from a smart environment by incorporating XGBoost for classification and ANN for consumption prediction. Alexiou et al. [22] modelled the sedentary behaviour of workers in a smart office using SVM, Naïve Bayes, CNN, and DNN algorithms. Jalal et al. [23] modelled the movement behaviour of occupants in a smart environment through signal denoising and linear SVM for classification. From all these behavioural studies, user modelling remains an open area for research because current behavioural modelling studies do not consider important user aspects. A user model acts as an information source about a user and comprises different assumptions about the behavioural aspects of the user. In the context of behavioural problems, a good user model provides an adequate understanding of human activities and provides insights into why an individual engages in an activity in a particular way, leading to the construction of an effective behavioural model.

In terms of intervention provision in smart environments, Ramallo-González et al. [24] developed educational interventions to minimise excessive energy consumption in a personalised and timed manner through the EnergyPlus software 22.2.0. LeBaron [25] developed the BESI-C system to collect behavioural and physiological data from a smart home and to provide interventions to minimise distress and improve self-efficacy in pain management for cancer patients. Two limitations were observed from these interventions in smart environments: (i) they do not design and deliver messages using preferences, likes, abilities, and disabilities specified within a user model, and (ii) they do not classify observed changes in behaviour before presenting the intervention. These limitations are the focus of the study.

The study will incorporate a new approach to behavioural modelling and intervention through the introduction of a novel behavioural model (PBC model). The PBC model considers, as input, important user aspects within a user model. Additionally, the PBC model classifies the observed changes before intervention provision. From the identified limitations, the questions that the study aims to answer are: RQ_1_: How can a behavioural model be developed to support positive behaviour change in a personal smart environment? RQ_2_: How can a user model be extracted from a dataset? RQ_3_: How can behaviours be modelled from user models? RQ_4_: How can a behavioural model classify human behaviours in personal smart environments? How can behaviour change be detected in Personal Smart environments? RQ_5_: How effective is the model in representing behaviours?

The study answered these research questions through the Design Science Research Methodology. The PBC Model was assessed through a naturalistic evaluation, which started with personal smart environment setup, daily activity engagements, data collection, data preprocessing, feature extraction, activity modelling, behavioural modelling, change detection, and change analysis. The significant contributions of this research are the formulation of the PBC model and the incorporation of the user model in behavioural modelling. The study is structured into the following sections: Section 2 will discuss related work in behavioural modelling using SEs. Section 3 will discuss the methodology; the associated theories will be discussed in Section 4. Section 5 will discuss the PBC model, Section 6 will discuss suitable application areas for the PBC model, while Section 7 describes the evaluation of the PBC model. The change-based interventions will be presented via a future publication. 

## 2. Related Work

The section will highlight existing works in user and behavioural models, sequential pattern mining, classification, change detection, and work productivity. These aspects are the techniques that will be incorporated into the study and are indicated in subsequent sections.

### 2.1. User Modelling

A user model is a source of information with many assumptions about the important behaviour of a system user [26]. In SEs, Gregor et al. [27] classified users into three categories in the disability spectrum. These are fit older people with no disability, older fragile people with few disabilities, and older people with disabilities that impair their functioning capabilities. These people depend on others to function well. User capabilities in this context refer to memory, learning, sensory, physical, elderly experience, and the environment. Additionally, as people age, there is a change in ability, including a decrease in sensory, cognitive, and physical functions, making user modelling a critical issue [27]. However, this categorisation is limited to the elderly; there is a need for a categorisation that will cater for all age groups. 

The proliferation of smart technologies has allowed the incorporation of the internet into our daily activities. User modelling in this era includes the description of the user’s context [28], such as observing a user’s attitude. With IoT, users can be understood differently through smart devices. Users’ internal states can be read, analysed, understood, and stored for numerous purposes [29]. As a result, user modelling goals have shifted from passive recognition to active creation because of the accessible collection of cognitive and behavioural information [30]. In the context of behavioural problems and monitoring, “a user model provides an adequate understanding of how users engage in behaviour, reasons for engaging in a behaviour in a specific way; and facilitates the building of an effective behavioural model.” For this study, the Generic User Model, which contains four sub-models, was proposed for this study in a previous paper [31]. These sub-models are: (1) the static user model, for unchanging information, for example, personal and role information; (2) the dynamic user model for changing information such as preferences, interests, likes, and dislikes; (3) the internal context information for health aspects of a user, such as physiology, emotion, cognitive and psychological aspects; (4) the activity model for relevant information that can describe a user or worker’s activities; and (5) the environmental context model for describing the physical features of a person’s environment and everything external to the person, such as the objects, sensors, and devices present. The current study will incorporate the user model presented in [32] in modelling human behaviour for the adequate interpretation of behaviour. 

### 2.2. Behavioural Modelling

Behavioural models are developed to describe human behaviours to assess performance and operation in a specific context [33]. Behavioural modelling helps to improve the functionality of SEs from a non-interactive environment to an interactive and real-time environment based on typical human control factors, thereby enabling decision-making when necessary. This includes people in the control circle because people are self-deterministic and love to be in charge of everything around them [34]. 

In modelling human behaviour within SEs, a large amount of data is needed to deduce, reason, model, and predict people’s behaviours. Human behavioural modelling is challenging because there is no availability of a complete physical model to describe possible behaviours. This is where data, knowledge, and hybrid modelling techniques come in. Conversely, no single method or model fits it all [34]. A behavioural model can be developed from the top-down by establishing associations between behaviours and tasks using knowledge-driven techniques. Additionally, a behavioural model can follow a bottom-up approach using data-driven techniques or combining both techniques through hybrid modelling. 

Regardless of the technique used, the primary goal is to generate the required outcomes [35] appropriately. These outcomes are the identification of good and poor behaviours and the actions/activities that create these outcomes. Therefore, if a behavioural model can recognise and predict human behaviour from a user model, then the patterns in the behavioural model should be classifiable for possible intervention provision. Different scholars have applied several techniques, but there are behavioural factors to consider for modelling. These factors are the contexts and sequential associations among human activities. Contextual factors include temporal associations and location, while activity associations refer to how people relate one activity to another [36]. These aspects are detailed in user modelling [31].

Tsai [37] listed three crucial steps for modelling human behaviours in SEs. These steps are knowledge discovery, activity extraction, and change classification. These three aspects must be incorporated to generate an effective behavioural model. Since human behaviour revolves around daily activities, knowledge discovery of activities becomes crucial, and this can be achieved through data, knowledge, or hybrid approaches. Irrespective of the learning technique, the goal is to differentiate good behavioural patterns from poor ones through activity extraction. Activity extraction deals with associating knowledge about how a user interacts with several activities in an SE to infer behavioural patterns.

Chen, Hoey, and Nugent [38] described a complex procedure for identifying people’s activities using SEs. These are the deployment of sensors in the environment for monitoring workers’ behaviour, collection, storage, and processing of sensed information for activity representation at a suitable level, the creation of computational models from sensed data, and the development and use of algorithms to infer activities. However, two issues arose from this procedure: (i) the absence of behavioural modelling inferences from the user model and (ii) the lack of behavioural classification for proper intervention/service provision. Langensiepen, Lotfi, and Puteh [39] followed this procedure to provide a model for office workers through data collection, data mining for recognising different worker features, and the use of recognised worker’s activities for building a worker profile, which was used to summarise worker activities and to adjust environmental conditions to suit a worker. Conversely, the worker’s model did not allow workers to change environmental conditions individually. Additionally, the worker’s model did not enable workers to adjust their resource usage in the environment. 

The behavioural modelling approach given by [37] is similar to the three behavioural modelling steps stipulated by [40]. Still, the significant difference between the two processes is the techniques used in each stage. Yu, Du, Yi, Wang, and Guo [41] specified three essential steps: data capture, modelling/analysis, and evaluation, which can be conducted using sensing devices, social media, and cameras. Modelling and analysis involve making certain assumptions about the data captured and applying these assumptions to understand human behaviour through an inference algorithm. However, a significant issue of this step is an inadequate theoretical demonstration that the model can understand human behaviour with the available data. Lastly, there was no performance evaluation of the model through universally accepted benchmarks; for example, metrics relating to efficient space and time can be used to evaluate a behavioural model [40]. This study will combine these approaches in modelling behaviour by developing and evaluating a behavioural model, specifically, the incorporation of sequential pattern mining for extracting behavioural models from user models in a subsequent section. 

### 2.3. Sequential Pattern Mining

Sequential pattern mining involves searching and extracting patterns from a database. Sequential pattern mining algorithms have been classified based on their search techniques, which can be breadth-first or depth-first [42] and their database format, which can be vertical or horizontal. Breadth-first algorithms use a level-wise approach by scanning a database to identify frequent sequences with a single item, then to find sequences with two items, and so on until there are no more frequent sequences. The search starts from the first nodes and moves to the next level nodes until it arrives at the root nodes [42]. Examples include Generalized Sequential Patterns (GSP), Prefix-Tree for Sequential Patterns (PSP), and the Apriori algorithm. Alibasa [43] used the GSP algorithm to mine frequent digital context patterns from digital technology usage logs for the mood prediction of workers. Depth-first algorithms identify recurring single patterns in a search space by starting with single items and recursively extending the initial frequent single patterns to create larger patterns. When a pattern cannot be extended, the algorithm reverses to identify other patterns using other sequences. Depth-first algorithms start their search from the first node and proceed to the next node in the same path until they reach the end node of the path [42]. Examples include Sequential Pattern Discovery using Equivalence classes (Spade), Prefix-projected Sequential pattern mining (Prefix Span), Sequential Pattern Mining (Spam), Last Position Induction (Lapin), the Co-occurrence Map (CM-Spam), and the Co-occurrence Map (CM-SPADE).

Based on the database format, algorithms in the horizontal family use a format where each row has a sequence identifier and an itemset list. For example, the Apriori and Prefix Span algorithms use the horizontal format for their mining operations. The vertical family algorithms use a vertical database format where sequence identifiers store patterns and the location of the pattern in memory. Such examples include SPADE, Prism, and CM-SPADE [44]. CM-SPADE, as a depth-first and vertical database format algorithm, was used in this research because CM-SPADE uses a vertical database format and a depth-first search technique, which allows fast processing. The CM-SPADE algorithm is an extension of SPADE: SPADE uses an equivalence class and sublattice decomposition to split the search space into several fragments. Each fragment then fits into the memory and is processed independently [45]. At the same time, CM-SPADE uses the basic implementation of SPADE. It also uses a co-occurrence map (CMAP) structure to maintain co-occurrent information for a database and for early filtering out of infrequent candidates to speed up the mining process. Additional parameters to the CM-SPADE algorithm are the support and window size values. These parameters are used to mine frequent item sets [46]. CM-SPADE was reported to be faster than previous sequential pattern mining algorithms. The strengths of CM-SPADE are the early pruning of candidate patterns, better performance because of low-memory requirement, more focused search space, and less candidate pattern generation than the breadth-first algorithms. In SEs, Suryadevara [47] demonstrated the effectiveness of the CM-SPADE algorithm in extracting residents’ behavioural patterns relative to their movement. The incorporation of CM-SPADE in this study is discussed in Section 8.4.

### 2.4. Classification

Behavioural pattern classification is often conducted via data-driven models. Bakar, Ghayvat, Hasanm, and Mukhopadhyay [48] classified data-driven models into supervised and unsupervised models. Supervised data models use the controlled learning approach, where labelled training data are made available for modelling. Examples include linear regression, Random Forest, Neural networks, and SVM [49]. These algorithms work by taking a known input dataset with identified outputs using trained algorithms to generate a reliable output prediction for a different dataset [50]. For the current study, Random Forest was selected as a suitable classifier for behavioural patterns.

The Random Forest algorithm belongs to supervised machine learning techniques. Random Forest is a hierarchical assembly of classifiers with the decision tree structure. Random Forest classifies datasets using a simple fixed probability to choose the most important feature during a classification task [51]. Random Forest reduces overfitting by merging several overfit evaluators to become ensemble learners. The behavioural pattern classification in this study is a multi-class classification problem where a behavioural pattern is classified into one of good, poor, or neutral. The application of Random Forest for behavioural pattern classification was motivated by the work of Khadse [52], where the authors incorporated the Random Forest algorithm for multi-class classification of an IoT sensor dataset with an accuracy of 91%. Furthermore, Alibasa et al. [43] incorporated Random Forest to classify digital behavioural patterns into mood classes with more than 75% accuracy. 

### 2.5. Change Detection

Change detection has been carried out via machine learning techniques, which can be offline or online. Offline change detection involves the use of historical data for change detection, and because of the requirement for a large dataset, a lengthy training period is required [53]. Offline techniques work by extracting models that describe the data and using these models to set thresholds for normal behaviour. The underlying assumption is that a dataset of a particular length is available. The goal was to ascertain a change in the series only after the data samples had been collected [15]. A significant limitation of offline techniques is dimensionality. As the data dimension increases, efficiency drops. Most real-world problems require that the change be detected in real-time, immediately or before a change occurs. Therefore, there is a need for unsupervised techniques. Examples of offline change detectors include Kernel Principal Component Analysis, Pelt search, outlier Dirichlet mixture model, binary segmentation, dynamic programming, one-class SVM, affinity propagation, k-means clustering, and the window-based search method [54].

Online change detection uses incremental learning, in which only data points within a window are used. In online detection, change is detected as new data streams continuously arrive. Online detectors run synchronously on a data stream by processing and identifying a change as soon as it occurs. As new data points arrive, online change detectors incorporate the new data points through knowledge retention rates for balancing previous and fresh data. With the new data points, new windows are generated and used. New window generation is usually accomplished by calculating the distribution’s probability/thresholds for the current run and updating the probability after another data stream arrives. Online change detectors can capture changes in real time because the probabilities/thresholds, which differentiate normal from change, can be frequently updated. When a change occurs, the probability reduces to zero [55]. Another way of detecting a change through online detectors is by comparing the most recent previous set and the next future set to decide whether there is a change [56]. 

Online change detectors have received much attention and have been adopted because of their ability to save computation cost, time, and storage time [15], thereby making online learners suitable for change detection. However, computationally, they are required to be fast and challenging to modify. Additionally, noise or unrelated data hinder online change detectors during training [53]. Online techniques include Self-Organising Maps (SOM) [57], bilateral PCA [58], Incremental Local Outlier Factor (LOF) [59], global LOF with local subspace outlier detection, Markov Chain modelling [60], K-means clustering [61], Single-Layer Feed-Forward Neural Network (SLFN), [57], Bayesian change point detection [62], Kernel Density Estimation (KDE) PCA [63], Generalised Likelihood Ratio Test (GLRT) [64], one-class support vector machine [65], and Isolation Forest [66]. This study used the Isolation Forest to detect productivity changes based on strength.

Isolation Forest is a collective approach used to identify changes/anomalies. It starts with a random feature and selects a partition between the highest and the lowest values to divide a sample. This process continues until the samples are divided. The Isolation Forest is constructed by including several isolation trees divided into several features. The number of divisions required to separate a sample is the path length from a root to a leaf [67]. Isolation Forest was established on Extra Trees [68], where each separation is random. A sample close to the root (i.e., short path length) can be distinguished effortlessly and is easier to separate from samples closer to the leaves. It is believed that change/anomalies will have a smaller mean path length than normal samples. When there is a sample at the leaf, the score will be close to 0. A shallow sample close to the root will have a score close to 1 [69]. The selection of the Isolation Forest algorithm for detecting changes in productivity was motivated by [70]. Isolation Forest works well for low-dimension data, even with unimportant attributes and contexts where anomalies or changes are absent in the training set [67]. Based on these capabilities, Shiotani and Yamaguchi [70] demonstrated the ability of the Isolation Forest to detect changes in patients’ physical condition (heart rate and dietary intake) in a smart care facility.

### 2.6. Productivity

Work productivity refers to the output produced with some input or resources. Often, productivity is defined as the ratio of output to input or resources used [71]. However, not all tasks are equal because some tasks do not require many cognitive resources and can be completed within a short time, while others may require much cognitive processing and may be completed in hours or days.

Within HCI, office work productivity has been studied by identifying factors affecting productivity. Specifically, social media usage, distractions, and interruptions have been identified and studied in detail. Borst, Taatgen, and van Rijn [72] studied work productivity by developing a computational cognitive model of task interruption and resumption by focusing on problem-state bottlenecks. The model was based on the memory for goal model, which states that when the central cognition queries the memory, the memory outputs the most active item at that instant. The memory for the goal model was extended by concentrating on the associated content of the problem state for each task through the memory for the problem state model. Czerwinski, Horvitz, and Wilhite [73] identified task complexity, the number of interruptions, task type, and length of absence as factors affecting workers’ return to tasks, while Mark, Czerwinski, and Iqbal [74] revealed that blocking online distractions was more beneficial to workers with low work control (i.e., self-control), low conscientious personality, and the absence of perseverance. In contrast to these studies, Kim et al [75] studied workers’ productivity in work and nonwork contexts and found mundane tasks and chores to be related to distraction and attention, as insufficient attention was associated with work productivity and intensity. The authors also identified several factors that affect productivity for the design of self-tracking applications. 

Office work productivity has been assessed through three methods: subjective, indirect, and direct methods. Subjective assessment involves the use of feedback from workers through interviews and surveys. Interviews are used for detailed study purposes and can only be effective when participants are few. A survey is used to gather data by sending questionnaires to many workers. This can be paper-based or through an online link sent via email or social network sites connected to an online survey database [76]. Sun, Lian, and Lan [77] used a subjective method to evaluate workers’ productivity through the self-evaluation of work performance and a fatigue scale to assess workers’ performance and productivity under ambient conditions. Chokka, Bougie, Rampakakis, and Proulx [32] also used the Work Limitations Questionnaire developed [78] to evaluate workers’ productivity. Workers’ productivity evaluation can also be undertaken through indirect methods such as worker’s absenteeism, number of grievances, number of hours worked, etc. These are usually accessed by another individual, usually the managers [79]. Data for indirect evaluation are obtained from the workers’ records, which usually include total time worked on a daily or weekly basis, output for a particular work duration, etc. [80]. Direct methods for productivity assessment are based on tracking workers’ digital device or software usage patterns [81] using tools such as screen life [82], Rescuetime [83], timeaware [84], and metime [85]. These applications enable the remote monitoring of workers’ activity through desktop/mobile applications installed on workers’ devices and phones. They continuously monitor workers’ activities by logging their activity data. From this list of applications, Rescuetime was chosen for this research because it offers more functionality than the others. 

The increase in the popularity of these tools has led to quantified self and lifelogging communities, where technology is incorporated into the data collection in a particular aspect of an individual’s life [86], especially work productivity. Using these tools indicates that productivity is a multi-dimensional concept. Users evaluate their productivity in six dimensions: work product, attitude to work, benefit, impact, workers’ state, and compound task. In all these dimensions, it was found that the quantity and quality of conceptual and concrete achievements were mostly considered work products [81]. These tools are limited to tracking simple activities, such as computer/phone usage and digital devices. They cannot track workers’ mobility patterns. This study followed a new direction in monitoring workers’ activities and estimating productivity using the PBC model in a personal SE that supports office tasks. Productivity was quantified by creating weights for each class that each behavioural pattern belongs to, multiplying the weight by the sequence length, and dividing by the sum of the weight. 

## 3. Research Design

The research aimed to develop and evaluate a new behavioural model for use in smart environments. The research followed a quantitative approach through experimentation to evaluate the model. The research design comprises the methodology and methods that were used to answer the research questions and achieve the overall objective of this research. These are explained in the next two sub-sections.

### 3.1. Methodology

The study incorporated the Design Science Research (DSR) methodology to design the PBC model. DSR involves scientific study and the creation of artefacts as people develop and use them to solve common practical problems [87]. In DSR, artefacts can be constructs, models, instantiations, and methods. Human behavioural studies focus on human behaviour in a context by developing and evaluating theories that seek to explain how and why an individual behaves to reflect the presence of problems. The goal of DSR is to create novel artefacts to solve problems and to ensure that the artefacts can extend the knowledge base of the problem domain [88].

Humans perform their activities in an environment. There is a continuous interaction between humans and their environments. When people behave, they interact with objects in their environment. These objects can be simple tools, technical devices, or systems. Therefore, people cannot be separated from their environments because they depend on the objects in the environment for task execution. The incorporation of DSR to model construction in human behaviour helps to guarantee the synergy between humans and their environment (as a usage context). DSR ensures that the model becomes useful for people and the environment for which it is created [89]. This study developed and evaluated a model as an artefact that can be used for promoting positive behaviours in SEs across several domains. The research falls into model development and evaluation, a core aspect of DSR, in which there are guidelines and activities for conducting model development. The important DSR activities in model development include problem identification, solution objective definition, model design and development, solution demonstration, solution assessment, and communication [89].

A strength of the DSR methodology is the presence of three cycles to enhance a researcher’s understanding of the conduct of high-quality DSR research. The cycles are the relevance cycle, rigour cycle, and design cycle. These cycles evolved due to the initial perceptions of DSR as either an artefact, which focuses on developing new products [90], or design-theory oriented, which focuses on developing new theory as an essential output of DSR [91]. Gregor and Hevner [88] suggested including these two approaches and a good balance between them. Therefore, any research utilising DSR should provide, as outputs, an artefact, and a theory behind the development of the artefact, using the three DSR cycles.

The relevance cycle focuses on understanding the problem and its domain, the requirements to solve the problem, and the opportunities in a specific environment to solve the problem. The domain is made up of people, organisational systems, and technical systems, which are used to recognise opportunities that can be used to solve the problem. When the problem and opportunities are recognised, the requirements for the relevance cycle can be easily defined, leading to the conversion of problems into requirements and objectives of the artefact. DSR also provides the criteria to accept a solution through evaluation by initially identifying the expected functionalities of the artefact and how the artefact in a domain can be improved [92]. The evaluation output will dictate whether further iterations should be performed within the relevance cycle or stopped. 

This study incorporated the relevance cycle through the proper problem statement definition of behavioural problems (Section 1), requirements engineering, and identifying opportunities to solve the problem (Section 2.2). The relevance cycle ensures that the personal SE is identified as an application domain. The personal SE is made up of continuous cooperation between organisational systems, people, and technical systems. The organisational systems in this context refer to the organisation’s structure. The organisational system can be functional, divisional, flat, or matrix. The people are the users of the SE. Technical systems refer to various systems or objects present in the SE that enable an individual to perform daily activities. The technical systems can be software, hardware, and IoT devices. The problem and opportunities for solving the problem are identified in the relevance cycle. For this study, the identified problem was behavioural problems. The solution opportunity identified in this research was behavioural modelling through personal SEs. The recognised problem and opportunities are used to describe the requirements needed for the relevance cycle. The requirements for the solution are the artefact’s ability to identify behavioural patterns and classify them into good, poor, or neutral. Additionally, the acceptance criteria for evaluating an artefact were created. If the model can identify and classify behaviours, then the artefact can be considered successful. The criterion became the output of the relevance cycle. 

The rigour cycle guarantees a meaningful association between past knowledge and new research by justifying its innovation and contribution [93]. Hevner et al. [94] recommended in-depth reviews of the existing literature to ensure that the contributed artefacts are not duplicates of previous artefacts. Therefore, the rigour cycle is based on the proper selection and incorporation of existing methods, literature, and theories to form a portion of the domain knowledge, develop a new artefact and assess the artefact. The newly created artefact will help expand the knowledge base of the problem domain, which is a significant goal of DSR [88]. The study used the Social Cognitive Theory and the SmartWork model. These are existing behavioural theories and are discussed in Section 4. 

The design cycle is the centre of DSR research [92]. According to Apiola and Sutinen [95], it is a construct–evaluate cycle for solution development because the design cycle iterates in artefact building and evaluation [93]. These iterations can happen more frequently than the rigour or relevance cycles. Major activities in the design cycle are artefact construction and artefact evaluation to check whether the requirements are met. The design cycle uses the outputs from the relevance and rigour cycles as input. These outputs are requirements, evaluation techniques, methods, and theories. However, maintaining a balance between rigour and relevance cycles becomes an important issue. Balance maintenance will help the researcher focus on the research direction. Balance maintenance can be undertaken by ensuring that the problem definition at the relevance cycle fuels the discovery of existing knowledge, theory, or models at the rigour cycle. Therefore, existing theories must refer to the identified problem. 

The design cycle involves building and designing an artefact and evaluating the artefact using the criteria stipulated in the relevance cycle to evaluate the artefact. The evaluation can be carried out several times, using each evaluation result as feedback to refine the artefact. Across the three DSR cycles, the study ensured a balance by using the knowledge base relevant to the problem domain. The study used requirement analysis and existing previous theories to develop a behavioural model in Section 5. The study also evaluated the behavioural model through a naturalistic-summative evaluation in Section 7.

### 3.2. Methods

The following methods were used for the research.

#### 3.2.1. Literature Review

A literature review revealed current user problems and available solutions using smart environments. With the literature review, gaps in the existing solutions were identified. The identified gaps enabled proper problem identification and motivation, as the relevance and design cycles of DSR stipulated. The output of problem identification is a problem statement, and the design of the PBC Model, with the main goal of answering RQ_1_.

#### 3.2.2. Experimentation

An experiment was conducted to answer RQ_2_, RQ_3_, RQ_4_, and RQ_5_. These RQs focused on how well the PBC Model identified the user model, behavioural patterns, and behaviour classification. The experiment evaluated the PBC model, as specified by the design cycle of DSR. The study experimented in a manner that allowed the capturing of behaviours from personal smart environment users. The environment included Rescuetime as a soft sensor and Fitbit as a hard sensor to capture users’ daily work activities and their heart rates while working. The details of the experiment were specified within the discussion on naturalistic evaluation. 

### 3.3. Sampling Criteria

The study included the following criteria for volunteers to participate in the study.

- Age must be minimum of 20 years;
- Must be computer literate;
- Must be users of desktop and smartphone;
- Must not have any impairment or special needs;
- Essential experience in computer and internet usage;
- Must be non-teaching staff;
- Must not belong to a population with special needs.

The data collection method for the research followed a quantitative method through experimentation. The experiment and procedures for the data collection and analysis are described in Section 7.

## 4. Theoretical Background

With the incorporation of the rigour cycle of DSR, the following theories were consulted and used in the formulation of the new behavioural model. 

### 4.1. The Social Cognitive Theory

The Social Cognitive Theory (SCT) states that the interaction among current behaviours, personal (affective and cognitive influences), and the environment determines an individual’s behaviour, mutually called “Triadic Reciprocal Causation” [96]. The SCT specifies the three determinants that interact with and affect behaviour. These are personal (cognitive, affective, and biological events), behavioural, and environmental factors [97]. A typical interaction is when personal factors control how people reinforce and model behaviours from others in an environment; these behaviours, in turn, control behaviours that people display in specific situations.

Environmental factors within the SCT came from the Social Learning and Imitation Theory (SLI) [98], which states that people are stimulated to behave in response to different responses, cues, rewards, and drivers from the environment. A very direct and recent precursor of SCT is the Social Learning Theory by [14], which describes how people learn through social methods of observing, replicating, and displaying the behaviours of others from the environment. The major effect of the environment on behaviour is social influence through behavioural mimicking in the environment. Learning as a social process signifies the primary goal of SCT and suggests that knowledge and skill acquisition comes from observational learning from role models within the environment. The mastery of new knowledge and skills is of higher interest than the object or the result of the learning process [97]. Therefore, the existence of humans depends on the repetition of behaviours from others in an environment with no impediments and when self-efficacy is high. Saleem, Feng, and Luqman [99] used the SCT to reveal that excessive social network use elicits cognitive–emotional preoccupation, which leads to poorer job performance in office workers. They also revealed that cognitive–emotional preoccupation increases task, process, and relationship conflict in offices as stressors from excessive use of social networks.

### 4.2. The SmartWork Model

Kocsis et al. [100] introduced the SmartWork model to assess aged workers’ health, emotion/stress, cognitive, task models, and workability. Workers’ health, emotion/stress, cognitive, task models, and workability were modelled from the remote monitoring of workers’ activities to evaluate their cognitive and functional decline by their employers for decision-making. The proposed SmartWork model comprises unobtrusive sensing\worker-centric block, a workers’ block, and the smart services block. The unobtrusive sensing framework is equivalent to the SE concept, with the functional purpose of collecting data relating to the worker’s physiological, lifestyle, and contextual aspects. The worker-centric AI block is equivalent to the user model, comprising a workability model, computational models, a self-adaptation model, and a dynamic simulation tool. 

The workability model was designed to extract workability from the heterogeneous data within the sensing framework. The workability estimate determines whether a person is entirely disabled, partially disabled, or fit for work. The computational models were designed to model a worker’s functional, cognitive, workability, and work task models. The self-adaptation model was developed to enhance a worker’s self-adaptation and management from the computational models. The dynamic simulation is a tool to allow training, decision support, intervention and on-the-fly resilient work management, work stress coping, and workers’ training. The AI decision support allows efficient decision-making based on the workability model. It aids work completion and team optimisation through nonrigid work practices. Risk assessment allows the specific assessment of risks that are related to training and health. It also allows adequate decision support and intervention provisions for employers or top managers. The smart services were designed to deliver smart services through continuously reporting workers’ health and lifestyle. 

Despite the high potential of the SmartWork model, several limitations were observed: (i) The narrow scope on older workers limits the model. A good model should be functional for all age groups in any SE. (ii) The worker-centric AI block was designed to model worker-specific parameters, but there was inadequate capturing of worker-specific parameters into the suitable models. For example, the worker’s functional, cognitive, workability, and work task aspects were captured within the computational models of the worker-centric AI block. 

These limitations were resolved by adequately structuring user-specific parameters into appropriate user model components, as undertaken in our previous work [31]. Other limitations are (i) the inclusion of the AI decision support and risk prediction models in the worker-centric AI block is inadequate. These models are services to be provided to top managers or employees to help them determine a worker’s state before assigning tasks to them. Therefore, better structuring of top managers’ services is recommended but out of the scope of this research. (ii) In terms of implementation, there was an absence of behavioural modelling, implying that the service provision was based on user models. Additionally, the absence of behavioural modelling implies no behavioural classification before service delivery. These limitations can be resolved by incorporating the behaviour model and classification as core components. (iii) The SmartWork model focused on modelling workers’ workability through score allocation but not on improving workability or productivity through services. (iv) The service component of the SmartWork model focuses on providing reports to the manager or the worker to determine the worker’s workability and to foster a manager’s decision-making concerning the assignment of a new task to a worker. 

These limitations can be resolved by including change-based interventions to improve individuals’ behaviours. The limitations in the SmartWork model led to the formulation of the PBC model, which is discussed in Section 5.

## 5. The Positive Behaviour Change Model

The SCT and the SmartWork model provided a theoretical grounding for developing a new model. The SmartWork model is the only extensive model in SEs that caters for aged workers’ workability, and it is similar to the proposed behavioural model in this research. However, these aspects were well differentiated and structured into appropriate models in the following ways: (i) Workers’ health modelling was modelled within the worker-centric AI block of the SmartWork model [100]. This study modelled health as an internal context within user modelling based on a previous study [31]. (ii) Cognitive modelling was modelled within the worker-centric AI block of the SmartWork model [101]. In this study, human cognition was modelled using the internal context within user modelling based on a previous study [31]. (iii) Emotion and stress modelling was within the worker-centric AI block of the SmartWork model [101]. This study modelled emotion and stress using the internal context within user modelling based on a previous study [31]. (iv) Work task was modelled within the worker-centric AI block of the SmartWork model [101] through work task requirements and task history. In this study, a work task was modelled as an activity, and activity modelling existed as a separate contextual entity within the developed Generic User Model [31]. (v) Services were provided as separate components and structured according to the purpose they were designed to meet. These services include care provision, teamwork management, and guidance provision. [101]. In the current study, services are equivalent to the interventions provided to users in a SE. Intervention exists as a significant component within the PBC model. However, the goal of the intervention component is to improve people’s behaviours in any SE.

From the limitations specified in Section 4.2, and the dissimilarities highlighted above, the Positive Behaviour Change (PBC) model was designed to promote positive behaviours in SEs, with the following assumptions: (i) User modelling is essential for behavioural modelling. A user model provides specific information describing the users’ static, dynamic, internal, external, and activities in their environment. (ii) Within the user model, there is a need for an objective assessment of activity models. (iii) There is a need for behavioural modelling from the user model, which will comprise behavioural patterns. (iv) Behaviour/behavioural patterns must be classified to identify the appropriate intervention type. (v) Sustainability is needed for the identified good behaviours and change for identified poor behaviours.

**Figure 1:**
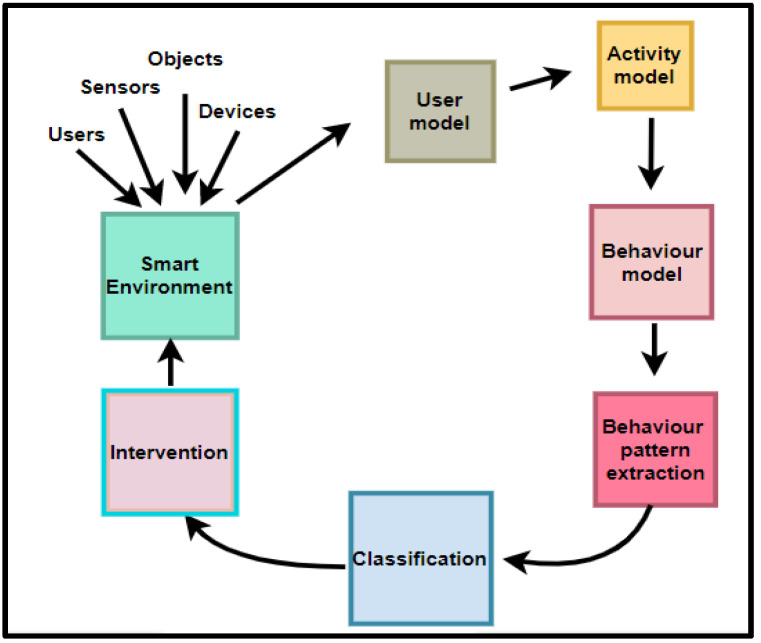
The Positive Behaviour Change Model v.1.

From these assumptions, this study proposes the PBC model, which comprises six components, as shown in Figure 1.

**Figure 2:**
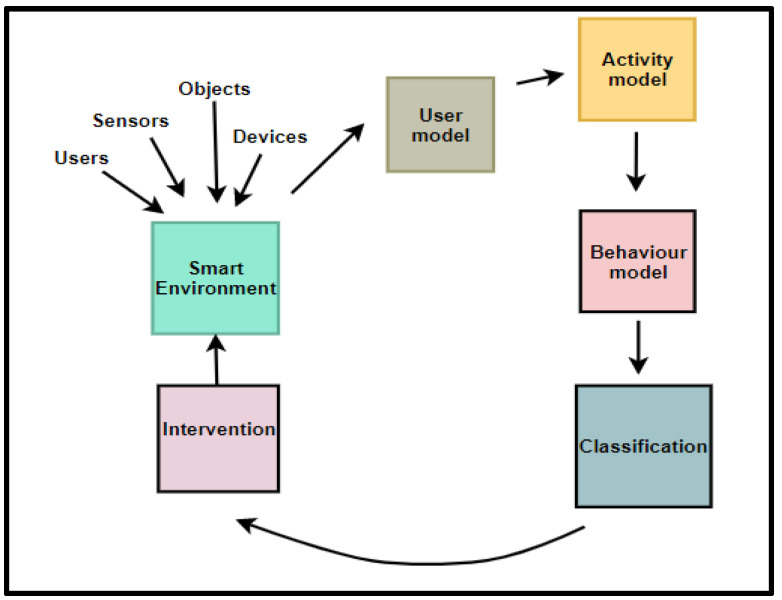
The Positive Behaviour Change (PBC) Model.

Communication is one of the activities in any research incorporating the DSR methodology. Therefore, the PBC model v.1 was communicated via a poster presentation. Feedback was received from reviewers. The feedback identified behaviour pattern extraction as a duplicate component because it is a core activity within the behavioural model. Therefore, behavioural pattern extraction was removed. The refined PBC model v.2 is given in Figure 2.

Figure 2 represents the condensed view of the PBC model, which is a model that focuses on modelling user behaviour and providing appropriate interventions to effect and sustain positive behaviours in SEs. The components of the PBC model are explained in subsequent sections.

### 5.1. PBC Model Components

The components of the PBC Model are explained in subsequent sections.

#### 5.1.1. Smart Environment

The SE can be a personal SE, a smart home, a smart office, or a building set up to provide personalised assistive services for its users through IoT and unobtrusive monitoring objects. The SE was included as a component in the PBC model because the SCT emphasises the importance of an individual’s environment on behaviour by positing that humans are active agents that influence the environment and are also influenced by the environment [96].

**Figure 3:**
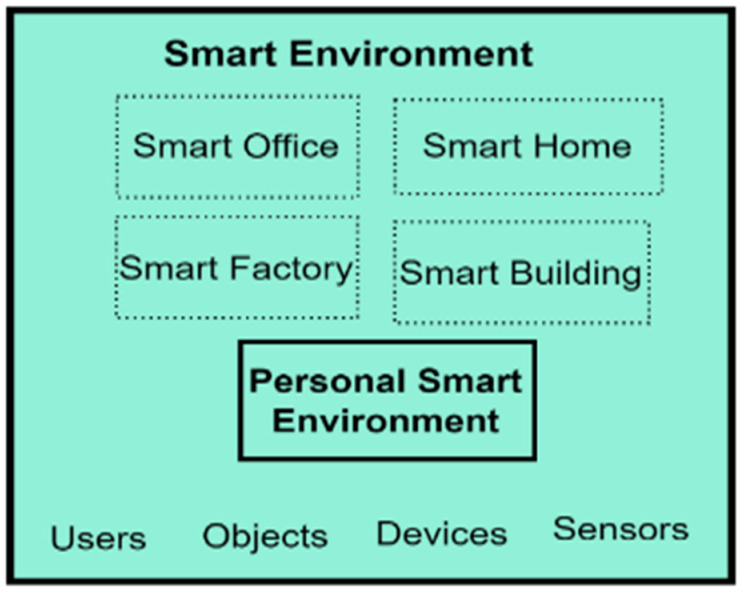
The Expanded SE Component of the PBC Model.

Additionally, the SmartWork model emphasises the inclusion of the SE through the unobtrusive sensing block, which comprises all smart devices that aid peoples’ task engagement and capture users’ lifestyle, physiology, and context information. The SE is a concept and an avenue for service delivery to humans. For evaluation purposes, this study used personal SEs, which allow official tasks to be carried out by workers. A personal SE was chosen because of its minimised procurement cost and easy setup in any natural environment. Furthermore, the performance of personal SEs is not affected by fixed spaces, making their services available in any geographic space in which the user is. Service delivery in fixed spaces is possible because the services developed for personal SEs are accessible via smartphones, making them available everywhere [102]. For this study, the digital activity data generated from the personal SE were used for building user models. The expanded SE component of the PBC model is shown in Figure 3.

#### 5.1.2. User Model

The user model provides information about users of SEs. The following theories motivated the inclusion of a user model in the PBC model. (i) The SCT emphasizes the effect of personal factors, such as cognitive, affective, and biological events, as determinants of behaviour [96]. (ii) The SmartWork model, where the health, emotion, stress, and cognitive aspects of workers were captured within the worker-centric AI block. (iii) In SE, Vlachostergiou et al. [103] emphasized the significance of the internal context of a user in producing observable behaviour. 

**Figure 4:**
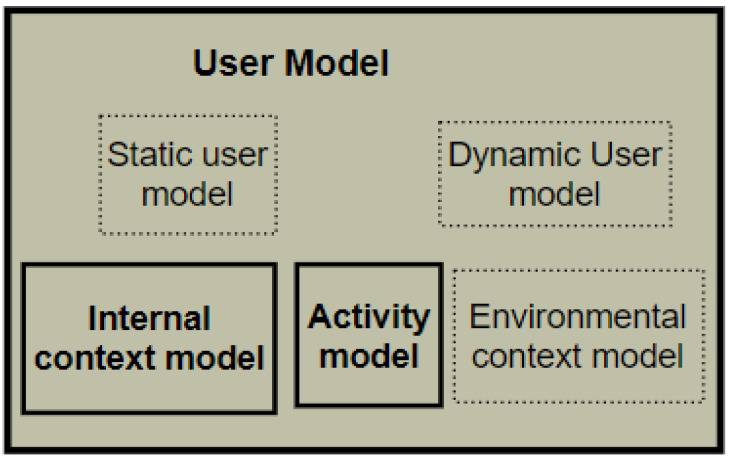
The Expanded User Model.

These are essential aspects of user modelling, but other aspects must be captured. In this study, the user model consists of several sub-models, such as SUM, DUM, internal context, activity context, and environmental context models. The details of these sub-models have been explained in a previous publication [31]. For this study, the specific output from the user model are the internal context and activity models. Users’ behaviours and behavioural deviation are built from the activity model, while the internal context provides an understanding of the users’ health and feeling during behavioural expression. The expanded User model of the PBC model v.2 is illustrated in Figure 4.

#### 5.1.3. Behavioural Model

**Figure 5:**
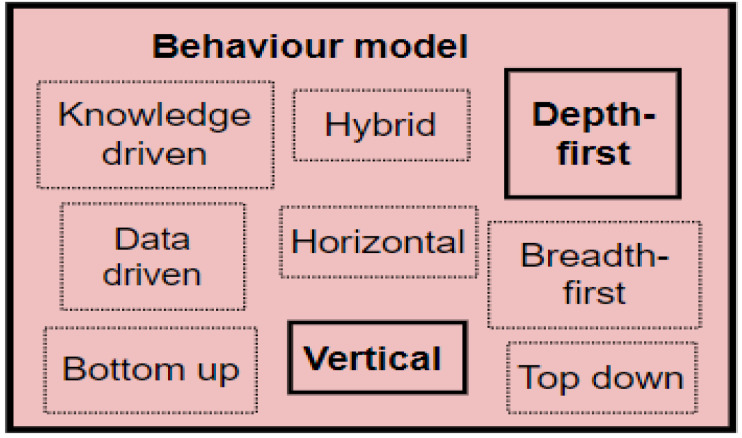
The Expanded Behavioural Model component.

The behavioural model recognises behavioural patterns from the identified activity sets within the activity model. The behavioural model is included because Patrono [104] emphasises the importance of behavioural models in SE for the early identification of risky behaviours. Section 2.2 identified knowledge, data, and hybrid approaches to behavioural modelling in SEs if the machine learning/data mining route is followed. On the other hand, the techniques can be breadth-first, depth-first, vertical, or horizontal approaches if the sequential pattern mining route is followed. The study followed the sequential pattern route in modelling behaviours. The expanded behavioural model of the PBC model is shown in Figure 5, while the behavioural modelling conducted in this research is indicated in Section 8.4.

#### 5.1.4. Classification

The Classification component is a module that classifies recognised behavioural patterns as received from the behavioural model. The last step specified by Suryadevara [37] was the inclusion of classification as a component. Previous models have focused on correcting deviations or changes from good behaviours through behavioural change detection techniques without proper knowledge of what a good or poor behaviour is, leading to minimal effects on people. In this study, a classification component was included because of the importance of classifying a behavioural pattern.

**Figure 6:**
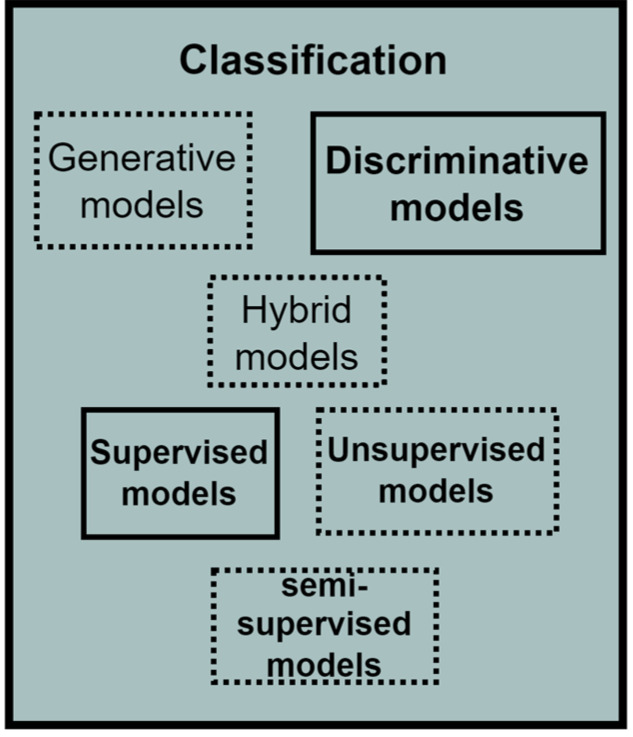
The Expanded Classification Component.

The classification component consists of several classifiers in the machine learning domain, which are usually data-driven. Depending on the probability distribution estimation, the classifiers within the data-driven category can be generative, discriminative, or hybrid. The data-driven techniques can also be supervised, unsupervised, or semi-supervised depending on the availability of label or class information in which the data or patterns are to be classified. The study incorporated a Random Forest classifier, which uses a discriminative approach in probability estimation and a supervised approach because of its requirement for labelled data. The expanded classification component of the PBC model is given in Figure 6, while the classification conducted in this study is explained in Section 8.5. 

#### 5.1.5. Interventions

**Figure 7:**
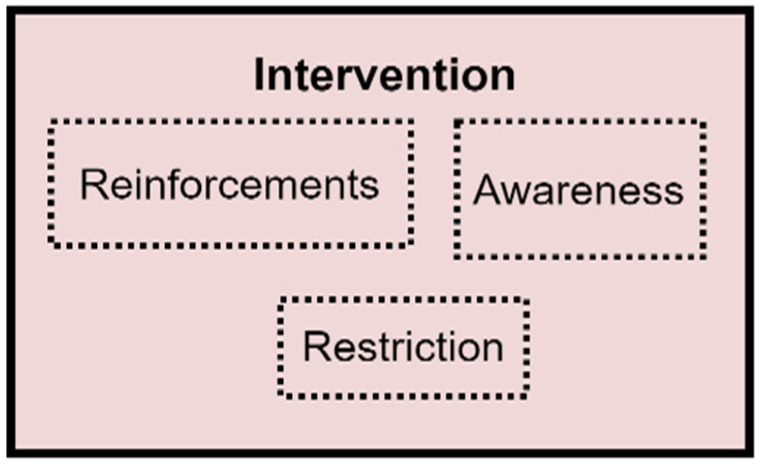
The expanded Intervention component.

The Intervention component implements interventions to be provided for the user, depending on the value of the quantified attribute evaluated and the change observed. Three interventions were identified to be appropriate for improving people’s behaviours. These are reinforcement, awareness, and restriction. However, these intervention options are not within the scope of this study, and they will be investigated in a future study. The expanded Intervention component of the PBC model is indicated in Figure 7.

**Figure 8:**
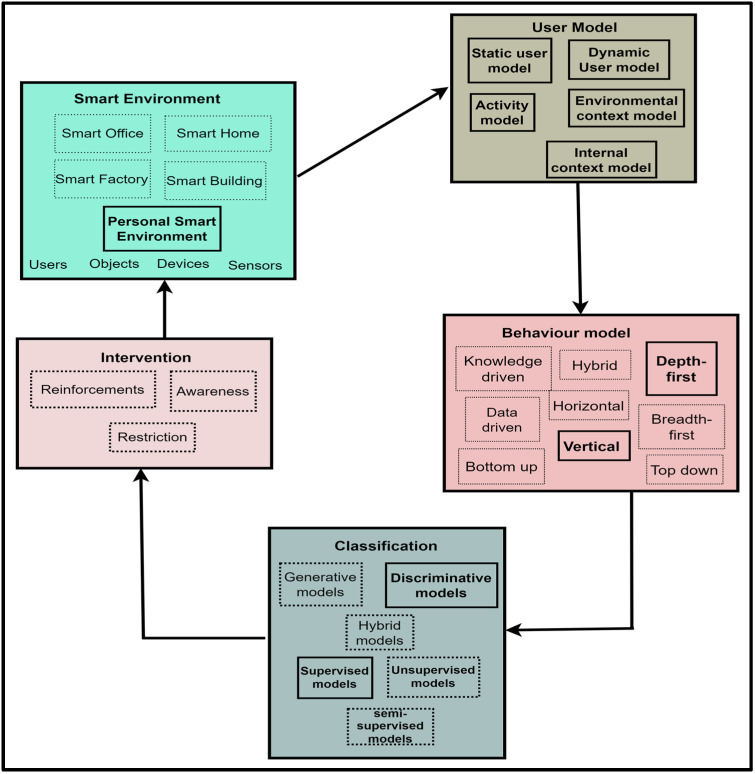
The Extended PBC Model.

From the extended PBC model components in Figure 2, Figure 3, Figure 4, Figure 5 and Figure 6, the Extended PBC model is represented in Figure 8 below.

Figure 8 represents the Expanded PBC model. Thick continuous lines represent the PBC model components incorporated in this research. Dotted lines indicate the unused subcomponents, which can be used for future behavioural modelling studies, depending on the specific aspect of SE users to be observed. The following points are to be noted by users of the PBC Model.

1. The components of the PBC model are not limited to the techniques specified within its components. Therefore, incorporators can use more advanced techniques that are suitable for the problem domain being studied.
2. In using the PBC Model, users can incorporate divide and conquer techniques, whereby the problem is divided into sub-problems according to the PBC Model’s components and sub-solutions are provided for the sub-problems. These sub-solutions can be combined to form the real solution to the problem under study.

Therefore, the RQ_1_ was answered through the development of the PBC model, comprising a smart environment, user model, behavioural model, classification, and intervention as core components required for behavioural modelling.

### 5.2. Model Interactions

**Figure 9:**
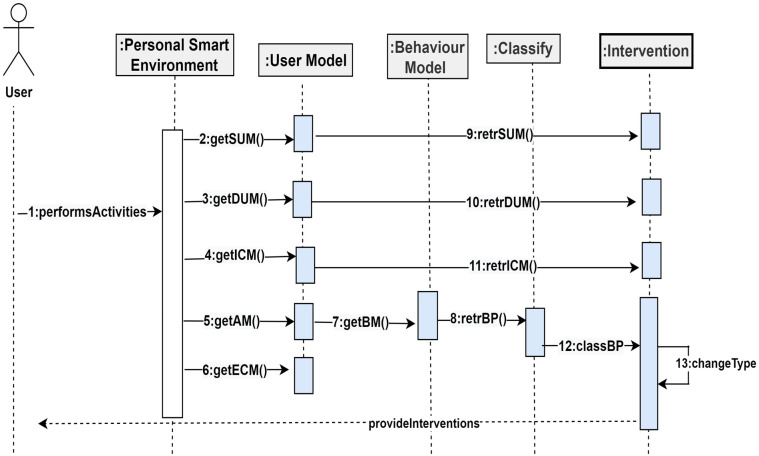
Component Interactions.

The components of the PBC model will interact with each other. These interactions are depicted in Figure 9 below.

Figure 9 shows the various interactions among the components of the PBC model. From Figure 9, a user in a smart environment can perform activities, which will be captured by the sensors in the smart environment, together with personal, role, preferences, likes, dislikes, personality, cognitive, psychological, health, and environmental attributes. These attributes will be modelled by the functions getSUM(), getDUM(), getICM(), getAM(), and getECM() within the environment to extract the static and dynamic user models, internal context, activity models, and the environmental context models. From the activity model, the function retrBP() constructs behavioural patterns from the activity sets. Additionally, the function classBP() will classify the behavioural patterns, which are used for change detection through the function changeType. The intervention component will determine the kind of change and returns change-based interventions to the user within the smart environment. 

## 6. Intended Use

The PBC model was developed to monitor and promote positive behaviours in any SE. The intended usage and typical use cases are discussed in subsequent sections. 

### 6.1. Work Behaviour Monitoring for Productivity

Section 1 highlighted multitasking/interruption, social media use, and low productivity as specific behavioural problems in offices. Therefore, workers must be monitored for these behaviours using smart devices in office environments. Attaran [105] emphasized providing stable workplace services with positive user experiences associated with people’s work. These user experiences can be obtained through worker engagement that will aid in the timely completion of tasks and faster achievement of work outcomes to increase productivity. These goals can be achieved by incorporating the PBC model into smart workspaces through constant modelling and monitoring to ensure the timely completion of tasks and by minimizing unnecessary engagement in behaviours that can reduce productivity. Using the PBC model will help workers be cognizant of productive and non-productive work behaviours. This section will not provide typical use cases for this intended use because the domain was used to evaluate the PBC model in Section 7.3.

### 6.2. Patient Monitoring in Smart Healthcare

Smart healthcare is an essential aspect of health that drives good healthcare delivery. Smart healthcare integrates information from different areas of patients’ lives to present an all-inclusive view of patients’ health in real-time [106]. Good healthcare services and resources can be delivered through the PBC model to achieve meaningful health improvement. Specifically, the behaviours of patients admitted to a smart healthcare facility can be continuously monitored for vital signs and to check for compliance to behaviours that will promote quick recovery. Such behaviours are adherence to drugs, timely breakfast/lunch/dinner, adequate sleep, and physical activity where applicable. The identification of sudden deterioration in these aspects can help healthcare workers in the determination and recommendation of appropriate services. This section will not provide a typical use case for this intended use because the domain is similar to health monitoring in smart homes or offices, which is discussed in Section 6.3.

### 6.3. Health Monitoring in Smart Homes or Offices

Taiwo and Ezugwu [107] advocated for a smart healthcare support system in homes to capture and record user health parameters. Within the smart health care support system, pulse, weight, blood pressure, glucose level, and body temperature can be the parameters for proper health modelling. With these capabilities incorporated into a smart home/office, the PBC model can monitor the health of different categories of people at home or in the office. Therefore, the overall health of an individual can be modelled. Additionally, young adults battling behavioural problems such as smoking, drug use, and physical inactivity can be monitored. Older adults with age-related health conditions such as dementia can be modelled and monitored. If there are deviations in health, a family member or a health caregiver can be contacted. A timely reminder can be sent for medication taking based on the information within the user model. The reminder can also motivate individuals for timely engagement in positive behaviours to promote their health at home or in the office. The modelling of health behaviours through the PBC model can provide immediate actions through reflections on behaviours that promote or deteriorate good health and well-being. The following use case illustrates how the PBC model can be used for health monitoring at home.

**Model**: PBC model
**Actor**: User
**Environment**: Smart home
**Scenario**: A user living in a smart home has temperature, door and window, and motion sensors. Additionally, he uses a glucose sensor for glucose monitoring. There is a simple mobile app that allows him to enter his glucose levels after each test and his daily food intake. The mobile app is connected via Wi-Fi to the central display unit at home, where the user can visualise his food intake patterns.
**PBC model description**: The smart environment component of the PBC model shall supply the temperature, window and door opening, and motion data, while the glucose data will be supplied through the glucose sensors attached to the body. The user will input the glucose and food intake data into the glucose-tracking mobile app on her smartphone. A typical food intake for a day can be bread, noodles, rice, pizza, salad, pasta, and vegetables, but there may be other food intake data for previous days. The user model component of the PBC model will extract the SUM, DUM, and activity model from the food intake data with their calorie estimation. Additionally, the blood glucose levels will be extracted from the glucose mobile app, serving as an internal context (health) model. The behavioural model component of the PBC model shall model user behaviours from the activity model. For example, rice–pizza–bread can be a typical behavioural pattern with calorie estimation. The classification component shall model the glucose levels using standard estimates of blood glucose to determine normal and abnormal blood glucose levels. Therefore, the output of the classification component will be a list of normal and abnormal blood glucose values, with their associated behavioural patterns (food intake patterns) and calorie estimates. The blood glucose model and the associated behavioural patterns will be used by the intervention component of the PBC model to determine the kind of blood glucose intervention to provide to the user within the smart environment.

### 6.4. Resource Use Monitoring in Smart Buildings

Lazzari [21] suggested using behaviour models to extract resource consumption usage in homes or offices. With increasing cost and reduced availability, resources such as electricity and water are vital components of human life. The PBC model can model and monitor people’s behaviours while using these two essential resources. Resource-use behaviour in consumption patterns can be modelled by initially gathering resource use data from smart meters installed in a SE, extracting consumption patterns from the data, and appliance usage from the consumption data. These aspects are the specific behaviour model that the PBC model can provide. Checking for over-consumption of these resources and communicating the information relating to appliance usage to users to promote better consumption of these resources is necessary. Furthermore, the PBC model can be incorporated into resource use modelling by checking for negligence when users are away from the building—for example, checking whether switches or taps are turned off to avoid waste. The user model within the PBC model can be used to control environmental conditions, such that the devices adjust to user preferences and manage resource usage through threshold checking. The following use case illustrates how the PBC model can be used for resource use monitoring in a smart building, which can be a home or an office.

**Model**: PBC model
**Actor**: User
**Environment**: Smart home
**Scenario**: A user living in a smart home has motion, a smart kettle, MircoWave, humidity, temperature sensors, a smart meter, a sensor attached to a cooker in her home and a smartwatch. Sensors are attached to all appliances in the house. The smart home has a smart display, which is an interface for interacting with its inhabitants. The user supplies her personal information through the smart display. The interface shows the user’s personal health and profiles, all stored inside the smart home database. The smart meter is connected to all appliances to capture energy consumption from the appliances. The user performs activities such as cooking, cleaning, ironing, watching movies, and listening to music. During cold days, the user uses heaters to warm up her environment and to maintain a balanced body temperature.
**PBC model description**: The user model component of the PBC model shall extract SUM from personal details, DUM from previously observed attributes from the database, internal context from the smartwatch through the smartwatch’s mobile app, activity from appliance usage, and environmental context models from the environmental attributes captured by sensors and stored in a database. User activities can be modelled from appliance usage. A typical activity model can comprise an iron, cooker, TV, and other previous activities. The behavioural model component of the PBC model shall extract behavioural patterns from the activity model. For example, cooker–refrigerator–iron–TV and TV–cooking–ironing can be typical behavioural patterns, with appliance usage information to monitor energy consumption. The classification component shall model energy consumption data using behavioural patterns to determine normal and high consumption. This classification will be used by the intervention component of the PBC model to determine the kind of energy conservation intervention to provide to the user.

### 6.5. Cultural Heritage

Cultural heritage institutions specialise in preserving a society’s tangible and intangible assets. These can be in the form of culture, values, and traditions. The advances in IoT drive recent advances in cultural heritage institutions by developing creative ways to present cultural content to visitors [108]. With the implementation of IoT for smart cultural heritage homes, more personalised and flexible experiences that consider visitor profile (user model) and behaviour are in high demand concerning environmental factors and the content to be presented to visitors. The PBC model is at the centre of the requirements because of the essential components for understanding the visitors and their contexts. Incorporating the PBC model will maximise cultural user experiences based on visitors’ preferences, interests, and capabilities. Other domains in which the PBC model can be used are students’ performance modelling in smart learning environments and consumer behaviour modelling in smart shopping environments. The following use case describes how the PBC model can be used in the cultural heritage domain.

**Model**: PBC Model
**Actor**: Visitor
**Environment**: Smart Museum
**Scenario**: A smart museum has exhibition displays, a touch screen, cameras, motion, temperature, beacon (a location sensor). A visitor has a smartphone, which is Bluetooth enabled. For a regular visitor in a smart museum, his face and location information will be tracked through sensors. On the first visit, visitors will supply personal information, preferences and personality, and cognitive-related information through the touch screen hung on the museum wall. Visitors move to several sections of the smart museum to view different exhibitions through the exhibition displays.
**PBC model description**: The user model of the PBC Model shall extract SUM from personal details and DUM from previously observed preferences and likes. Internal context can be extracted through the personality and cognitive information supplied by the visitor. The user model component of the PBC model will extract the activity model from the location sensor. For example, a typical activity model can consist of art, culture, history, science, and war. The behavioural model component of the PBC model shall extract behavioural patterns from the activity model. For example, an extracted behavioural pattern can be war–science–culture–art. There can be a typical behavioural pattern with higher frequency for a visitor. This pattern can be used by the classification component to detect changes in exhibition views, such that the content of the displays can be updated in real-time to reflect the change for the next exhibition to be viewed or for future visits. Furthermore, a visitor close to a location sensor connected to a smartphone via Bluetooth can receive exhibition messages so that the visitor can be aware of the various exhibitions available and advice regarding navigating through the museum.

This study used productivity modelling and monitoring to evaluate the PBC model, as discussed in subsequent sections.

## 7. Materials and Methods

The study focused on behavioural modelling and monitoring in SEs, specifically using office environments that support smart objects. The evaluation aims to demonstrate the PBC model’s ability to model human behaviours through pattern recognition and classifying the identified behavioural patterns. Therefore, the study evaluated the PBC model through an experiment using office environments with personal smart devices installed. Therefore, personal smart office environments (PSOE) were used. A personal smart office environment was chosen because of the following reasons: (i) There is only one full smart environment in the research institution. (ii) The participants cannot be moved from their natural working environment to the university’s SE, as this will introduce some biases into the study design and responses. (iii) Full SEs cannot be implemented in each participant’s office due to cost implications. (iv) PSOEs can be set up in each participant’s natural offices with minimal costs using simple sensors. The experiment is described in subsequent sections. 

### 7.1. Evaluation Objective

The evaluation objective of this study was to instantiate the PBC model as an artefact produced from DSR and to evaluate the core components of the PBC model in a naturalistic environment.

### 7.2. Evaluation Process

The evaluation steps specified in the Framework for Evaluation in Design Science (FEDS) by Venable et al. [109] were adopted to identify the evaluation goals, select suitable evaluation technique(s), decide on the properties to evaluate, and plan each evaluation episode. These steps are summarised in Table 1.

Table 1 shows the evaluation process that was followed by the study. Step 1 focused on identifying the evaluation goal, which mapped to rigour evaluation, where the evaluation ensures that the artefact is usable in a realistic environment (office environment). In step 2, the human risk and effectiveness strategy was chosen because it helps to evaluate an artefact’s effectiveness through a naturalistic evaluation. In step 3, the evaluation properties for evaluating an artefact were selected. These are user model creation, behavioural pattern extraction, behaviour classification, and change detection. In step 4, the plan for the selection evaluation is decided. For the naturalistic–summative evaluation, experimentation with office workers was carried out. The details of the experiment are discussed in Section 7.3. 

### 7.3. Naturalistic-Summative Evaluation

The naturalistic–summative evaluation design incorporated the instantiation of the PBC model through the implementation of personal SEs in offices to collect the daily digital work data while at work. The evaluation was designed in a way that allowed non-intrusive data collection, i.e., as participants are working, the digital activities are logged without diverting their attention or disturbing them. The activity dataset included desktop/mobile phone and internet use, social media use, email, and interrupt/multitasking activities. Additionally, the study collected participants’ heart rate data as a vital sign and an internal context to reflect their health status while engaging in their office tasks. These datasets were collected for 4 weeks per participant, spanning from November 2020 to April 2021.

#### 7.3.1. Participants

Data acquisition for the study involved collecting digital activity data, acting as primary data. Before the commencement of data collection, Nelson Mandela University’s Ethics Committee approved the data collection (H20-SCI-CSS-006). Invitations were sent to the Physical and Support Staff (PASS) of Nelson Mandela University, South Africa. Within the invitation, the eligibility criteria stated in Section 3.3 were included for consideration and participation. 

Digital activity data were obtained from workers in offices with personal smart objects. Thirty invitations were sent, but only eight staff members responded and accepted to volunteer for data collection, while two staff members withdrew before the actual setup of the personal SEs. COVID-19 restrictions caused a low number of participants, reduced the number of times workers worked in offices, and discouraged physical contact and setup of devices in workers’ offices. The participants were females between 20 and 59 years of age (mean = 42). One participant was a secretary, four participants were administrators, and one participant indicated that she was a non-teaching staff member. No measures could be taken for the gender bias because they were the people who indicated an interest in the study. Before the commencement of the experiment, the volunteers were given consent forms. They agreed with the terms specified within the consent forms. Race/ethnicity was not collected because the experiment aimed to model their work behaviours in offices and not the impact of race/ethnicity on work behaviours or productivity. 

#### 7.3.2. The Dataset

Six participants volunteered to participate in the data collection, but 48,444 records were retrieved over a period of four weeks for 1080 h with a total number of 120 days, which is sufficient for most data mining and machine learning algorithms. The data collection aimed to build a user model that will provide relevant information about a user, which can be static, dynamic, or contextual. Two data types were collected. These were digital activity and heart rate data. The digital activity dataset was collected via Rescuetime [83]. The activity dataset contains the date, seconds, activity, category, and productivity data. Fitbit Charge 2 were used to retrieve participants’ heart rate data. The heart rate dataset contains the date and the heart rate data.

## 8. Results

For this study, exploratory data analysis, behavioural feature extraction, activity modelling, behavioural modelling, behavioural pattern classification, change detection, and analysis were performed on the dataset. The results from these activities are discussed in subsequent sections.

### 8.1. Exploratory Data Analysis

Exploratory Data Analysis (EDA) was performed to provide general insights into the dataset [110]. The data consisted of activity class as a categorical variable and heart rate as a numerical attribute. However, meaningful explorations were derived from the summarised heart rate and digital activity data.

#### 8.1.1. Heartrate Exploration

Descriptive statistics were used to summarise the heart rate data. The study used the mean and median as the central tendency measure and standard deviation as the dispersion measure to unravel the dataset’s distribution. The distribution of the heart rate data is shown in Table 2.

Table 2 shows the descriptive statistics for the heart rate values of all participants. The participants’ age range was 20 to 59 years. Generally, the mean heart rate range is 60–100 bpm, but for the age of this population, the mean heart range value was 70–75 bpm [47]. From Table 2, all the participants had mean heart rates higher than the specified mean heart rates for their age but were still within the range of the general mean heart rate values. From Table 2, P4 had the highest mean heart rate (89.00 bpm), and P2 had the lowest mean heart rate (66.52 bpm). Heart rate differences are specific to individuals. Heart rate cannot be generalized to an entire population because many factors can affect people’s heart rate, for example, age, gender, health, and exercise [107]. More in-depth analysis through change detection and correlation analysis was conducted in Section 8.8 to see whether there were associations between individual-specific heart rate values and productivity. Missing values for the heart rate data were checked and input from the normal distribution of the unmatched data based on the work of Pouri and Hilty [93].

#### 8.1.2. Computer Usage Exploration

**Figure 10:**
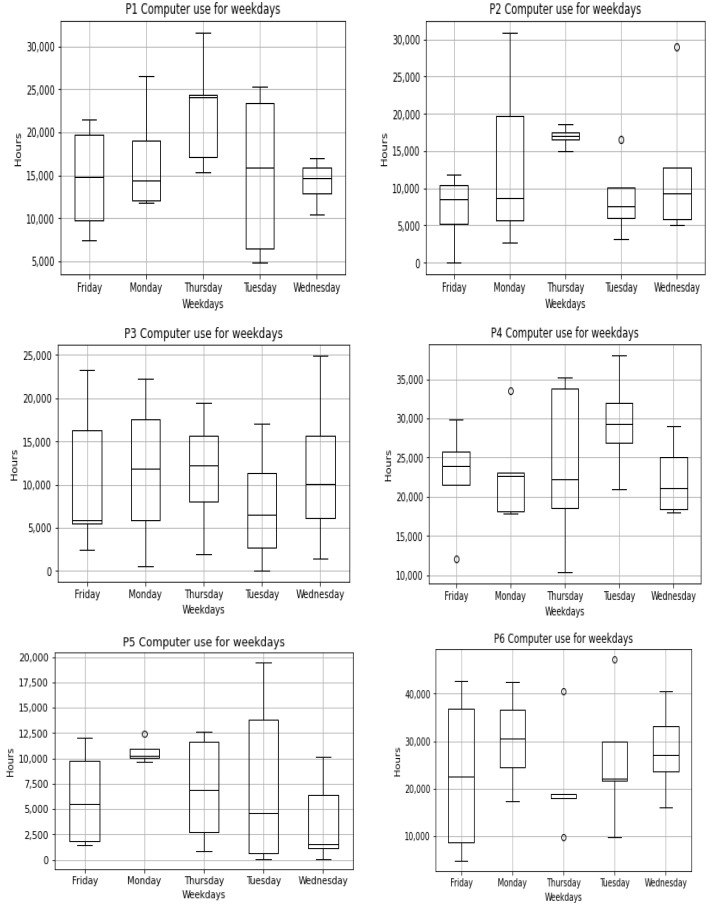
Weekday Computer Usage per Participant (*n* = 6).

The study explored participants’ computer use for each day of the week and hourly distributions. Computer usage per weekday is represented in Figure 10 and shows that there was a variation in computer usage on weekdays and weekends; participants’ computer usage was not similar. It was specific to everyone.

**Figure 11:**
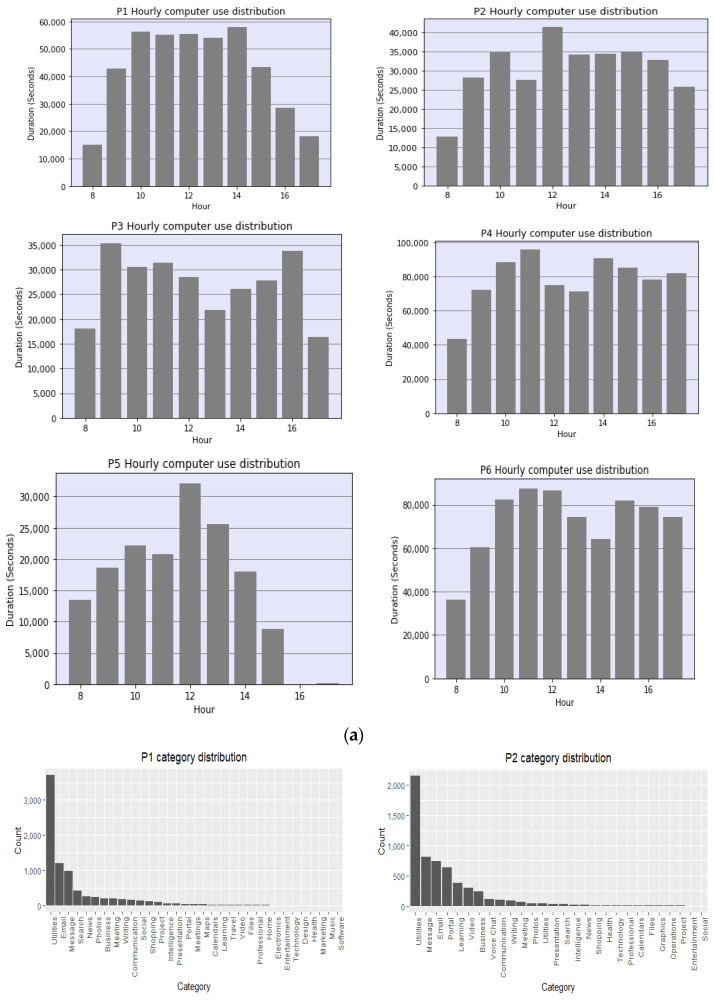

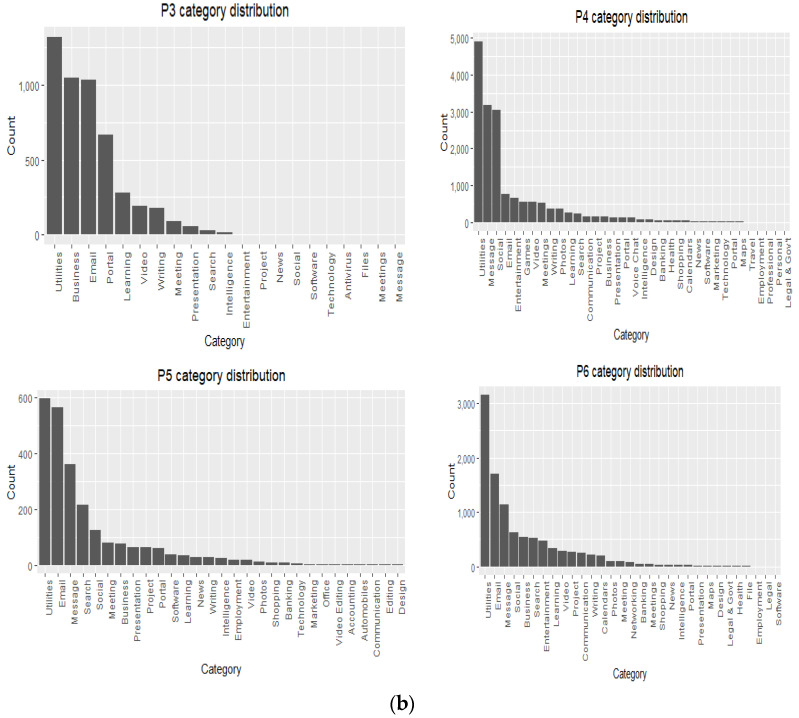
(**a**). Hourly Computer Usage per participant (*n* = 6). (**b**). Work tab engagements.

Figure 9 shows that P1 peaked on Tuesdays, P2 and P3 peaked on Mondays, P4 peaked on Thursdays, and P5 peaked on Tuesdays. P6 peaked on Mondays and Fridays. These observations were based on four-weeks worth of data from each participant. The hourly distribution is shown in Figure 11a.

Figure 11a showed that P1 had peaks at 10 and 14 h, P2 had a peak at 12 h, P3 peaked at 9 and 14 h, P4 peaked at 11 and 14 h, P5 had a peak at 12 h, and P6 peaked at 11 and 12 h per day over one month. The peak observations showed a general increase in engagement in the morning, while the peak hours varied from one participant to another.

#### 8.1.3. Work Tab Engagements

The study explored the participant’s work tab engagements by visualizing the most frequent work tab that each participant engaged with. Work tab exploration will provide insight into the frequent work tabs that dominated participants’ computer usage. This is shown in Figure 11b.

Figure 11b shows each participant’s work tab engagement in terms of the frequency of the tabs engaged with during the data collection period. Figure 11b shows that P1 engaged with Utilities, Email, Message, Search, and News. P2 engaged with Utilities, Email, Portal, and Learning. P3 engaged with Utilities, Business, Email, Portal, and Learning. P4 engaged with Utilities, Message, Social, Email, and Entertainment. P5 engaged with Utilities, Email, Message, Search, and Social. P6 engaged with Utilities, Email, Message, Social, and Business. These are the top five tabs observed for the participants.

### 8.2. Behavioural Feature Extraction and Event Log Generation

Behavioural feature extraction was further divided into data pre-processing, feature representation, feature selection, and behavioural pattern generation. Data pre-processing was carried out to ensure that outliers were not expunged because they were needed for change detection and analysis and to ensure proper representation that is adequate for machine learning algorithms. An event log was generated for behavioural modelling to overview workers’ events and how they perform their daily work activities. An event log is a set of events consisting of an activity (A), Timestamp (T) and event type (ET), which can be a start or complete [41].

**Figure 12:**
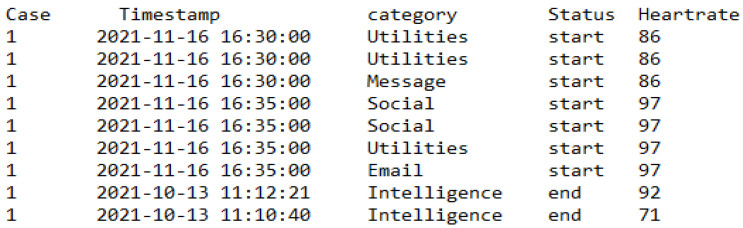
An Example of Event Log.

Data pre-processing, feature representation, feature selection, event log generation, and behavioural pattern generation were conducted by selecting appropriate attributes and converting the digital activity dataset into event logs to see how workers perform their tasks. The event log fetched the preceding and succeeding activities for activity modelling. These steps resulted in an event log that was ready for further analysis. Feri and Y. Bernardo [41] indicated the case, timestamp, activity column, and event status as the primary event log information. A case is an identifier for a person. Timestamp signifies the time of the event, activity/category is the event name, and status signifies what the timestamp represents. It can be the start or the end of an event. However, heart rate was added to signify internal contextual information. This information is shown in Figure 12.

### 8.3. Activity Modelling

The activity models were extracted from the event log using a heuristic miner. The heuristic miner is a process mining algorithm that extracts processes from an event log by considering the frequency of activity. The significant advantage of the heuristic miner is the ability to deal with nonlocal dependencies, short loops, and noisy data while extracting a correct model. Rojo et al. [111] incorporated a heuristic miner in the activity extraction of smart home residents, and it has been proven to be efficient in modelling smart home residents’ activity models. 

**Figure 13:**
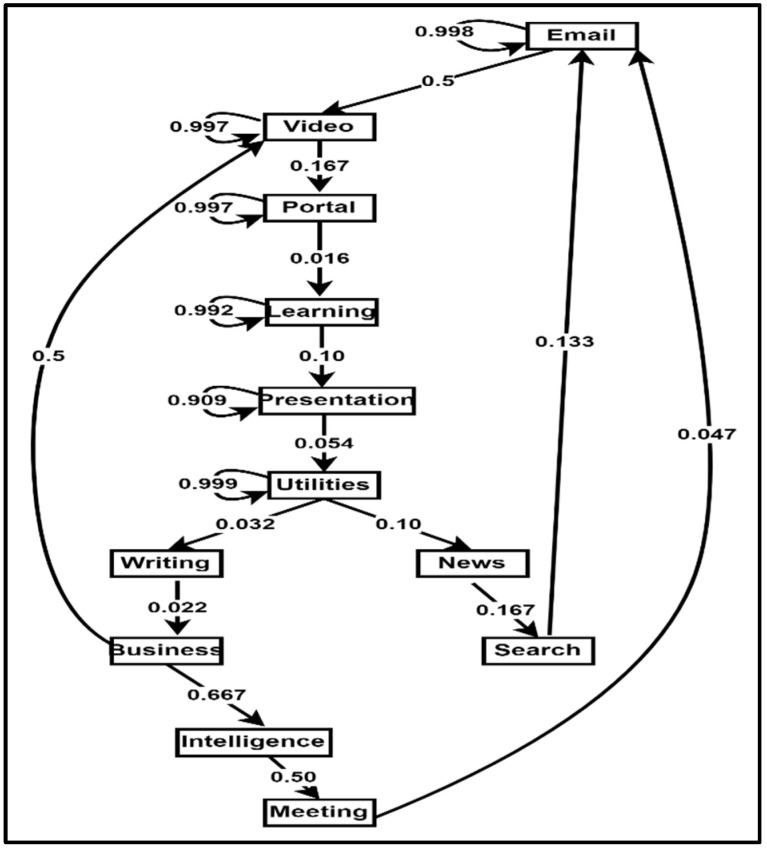
Activity model for P3.

In this study, a heuristic miner was applied to the event logs. The activity models were represented in dependency graphs, where the nodes signify activity categories, and the arcs signify causal dependencies between nodes. Causal dependency refers to how an activity depends on or leads to another. The arcs were annotated with weights, which signify the dominance of activity relations based on causal dependency between the nodes [112]. An activity model for a participant is shown in Figure 13. The study considered a weight of 0.4 in determining weighty paths because, at this value, all necessary weighty paths were captured. The weighty paths indicate the core activities and dependencies in each participant’s user model.

From Figure 13, P3 had the following weighty paths: email–video and business–intelligence–meeting. The logs from the activity models were used in behavioural modelling in Section 8.4. Therefore, RQ_2_ was answered through the extraction of activity models as a core component of the user model. Furthermore, the first evaluation objective (user model), as recommended by the DSR methodology in Table 1, was achieved.

### 8.4. Behavioural Modelling

This study followed a novel approach to behavioural modelling. Behavioural modelling was conducted from the user model, specifically the activity model, to create and group item sets based on temporal relations, identify behaviour patterns, classify behavioural patterns, behaviour change assessment, and make intervention recommendations. Item sets were created by encoding the categorical variables into numbers through a label encoder. The encoded categorical variables with the same timestamp were grouped. Item sets within a window of 30 min were grouped to form a sequence database. The sequence database was formatted into a basket format required by the CM-CSPADE algorithm. 

The participants’ behaviours were modelled using the sequence database as input into the CM-SPADE algorithm. Minimum support of 0.6 was used as an input into the algorithm to generate frequent patterns. Additionally, the encoded categories were formatted back to their standard text form. The behaviour modelling that was conducted through pattern extraction was specific to everyone. The behavioural model from the six participants cannot be generalized to the entire population of administrative workers because there were variations in how people engaged in their duties. However, for each participant, each behavioural pattern comprises several activities relating to work and nonwork activities. Table 3 shows the number of behavioural patterns extracted from each participant’s user model and each participant’s event log size.

**Figure 14:**
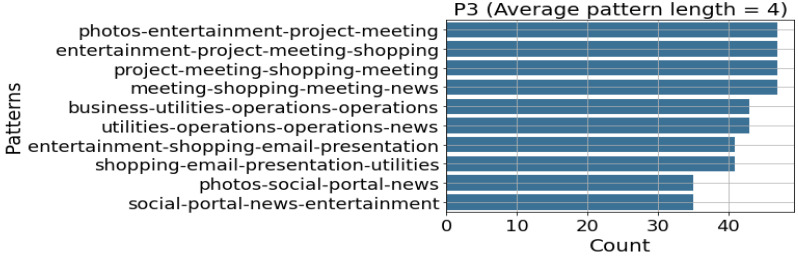
Top N-gram for P3.

Table 3 shows the number of behavioural patterns identified from each participant’s user model, with P6 having the largest event log and P2 having the largest behavioural pattern sizes. Therefore, the second evaluation property (pattern recognition) specified in Table 1 was verified by showing that the behavioural model component of the PBC model can identify behavioural patterns. Within pattern recognition algorithms, several conditions were stated and tested to ensure the validity of the identified behavioural patterns. These conditions include comparing the performance of an algorithm to other algorithms through computational time and memory usage [44,46]. However, the behavioural modelling in this study is not comparison-based because of the use of a single source of human behavioural data and the CM-SPADE algorithm only. Therefore, the focus was not on evaluating the performance of several algorithms on the participants’ dataset but on extracting behavioural patterns from each participant’s event log. Furthermore, n-grams were constructed to view the top behavioural patterns based on the mean pattern length for each participant, as indicated in Table 3. The top behavioural patterns are represented in Figure 14.

**Figure 15:**
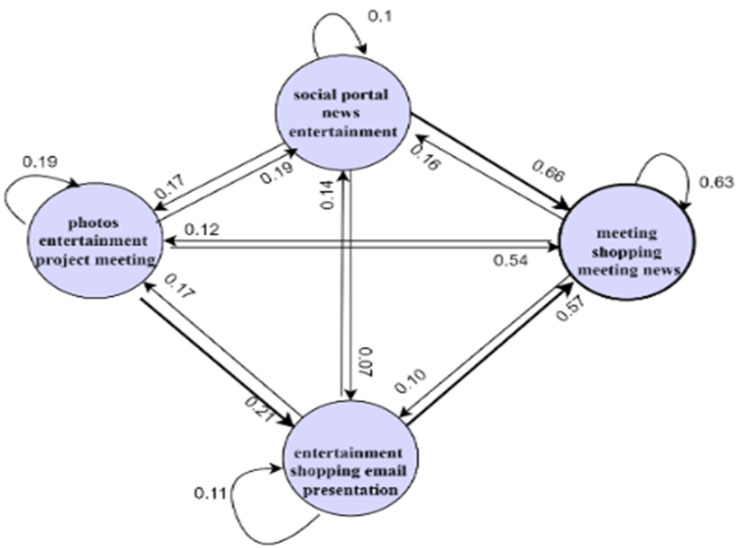
Behaviour model from P3.

Figure 14 shows the top patterns for P3 based on the mean pattern length. All participants’ patterns consisted of the activity “Utilities”. Utilities are sets of applications on desktops or laptops, which signify a core activity that must be engaged in for other activities to occur. Therefore, “Utilities” was a core component of the participants’ engagement in their activities. These top sequences were extracted from each participant’s behavioural pattern dataset. Their probabilities were estimated through Markov probability estimation, based on the work of Amato et al. [113], where social network behaviours were modelled through first-order Markov chains by computing the probabilities of transitions among nodes. The probabilities signify a state’s transition likelihood. In this study, first-order Markov chains were used to analyse participants’ transition from one behavioural pattern to another based on the mean sequence length for each participant. From Table 3, only behavioural patterns of mean length were considered to allow simplicity and understanding of the behaviour models. The behavioural patterns were represented as nodes, while the edges represent the transition probabilities in Figure 15.

From Figure 15, P3 had a mean behavioural pattern length of four, which was used to generate the graphical model. Four behavioural patterns were observed from the graphical model of P3, forming four states. All states were self-transiting, meaning that P3 maintained a behavioural pattern before switching to another pattern. All transitions to {meeting, shopping, meeting, news} have high probabilities, indicating that {meeting, shopping, meeting, news}is a core behavioural pattern for P3. P3 engaged more often in this pattern than others. The following paths (probability > 0.1) can be observed in Figure 13.

{meeting, shopping, meeting, news} -> {entertainment, shopping, email, presentation} -> {photos, entertainment, project, meeting} -> {social, portal, news, entertainment}

{meeting, shopping, meeting, news} -> {entertainment, shopping, email, presentation} -> {social, portal, news entertainment}

{meeting, shopping, meeting, news} -> {photos, entertainment, project, meeting} -> {entertainment, shopping, email, presentation}

{photos, entertainment, project, meeting} -> {entertainment, shopping, email, presentation} -> {meeting, shopping, meeting, news}

{social, portal, news, entertainment} -> {photos, entertainment, project, meeting} -> {entertainment, shopping, email, presentation}

{social, portal, news, entertainment} -> {meeting, shopping, meeting, news} -> {entertainment, shopping, email, presentation}.

Therefore, RQ_3_ was answered through the extraction of behavioural patterns from the activity models. Furthermore, the second evaluation property (behavioural pattern extraction) was achieved.

### 8.5. Behavioural Pattern Classification

**Figure 16:**
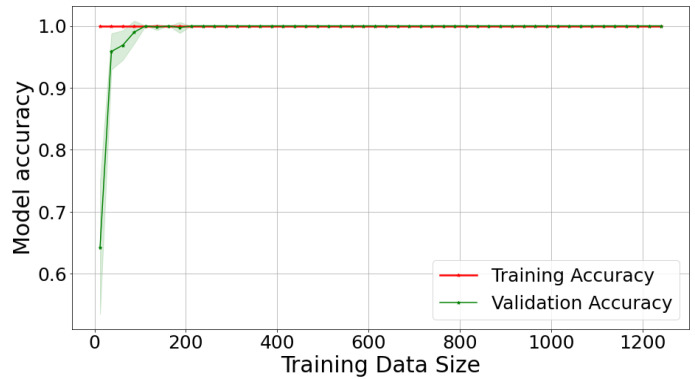
Learning Curve from P1.

Before conducting classification, sample size estimation for the Random Forest classifier was carried out through a learning curve to avoid over/underfitting problems and to determine the sample size at which the Random Forest algorithm model will reach its maximum accuracy. The study incorporated the learning curve interpretation provided by Chen et al. [114] to determine the point where the sample size of the dataset is adequate and to ensure that the dataset size is sufficient for behavioural pattern classification. The learning curve is shown in Figure 16.

Figure 16 illustrates the performance of the Random Forest classifier through the learning curve. Figure 16 shows that the training set maintained its maximum accuracy and the accuracy of the validation set improved with the increase in training samples. From Figure 14, the training accuracy and validation accuracy curves achieved the highest accuracy and converged when the training sample size was 150. From Table 3, each of the participant’s behavioural pattern samples was more than 150. Therefore, there are enough pattern sample sizes to train the Random Forest algorithm.

Behavioural pattern classification was based on a multi-class classification method, which involves classifying the behavioural patterns into good, poor, and neutral behavioural classes. A good behavioural pattern is a pattern that consists of work-related activities, while poor behaviour is the opposite of good behaviour. The behavioural pattern classification into good, poor, or neutral patterns used simple heuristics to identify the presence of good and poor items in a sequence. One hundred and forty-four entries of a participant’s behavioural pattern dataset were classified using a function that searches for a substring and assigns a tag to the pattern if the pattern was found in the string. For this study, a behavioural pattern is a string comprising several activity categories. A substring refers to an activity category or categories with lengths smaller than its parent string or behavioural pattern. A key is an item to be searched for in a behavioural pattern. Class balancing and adequate sample size were ensured through sampling techniques to improve the classifier’s performance. This study looked for social networks, news, opinions, blogging, and non-office-related activities. Therefore, any pattern that does not contain these items will be classified as *good*, and the presence of these items in any pattern will signify a poor pattern. However, some items cannot be classified as good or poor, as these items do not signify good or poor activity engagement, and the individual’s intention for these patterns cannot be ascertained. These include general utilities, browsing, and searching. The motive for these activities cannot be ascertained, and they were assigned the neutral tag. However, not all patterns were correctly classified by the function. 

The correctly classified patterns from the initial classification were used to classify other participants’ behavioural datasets using a Random Forest classifier. For the Random Forest classifier, the classification into a class was determined through voting by obtaining corresponding voting results of each decision tree and selecting the result with the highest vote [115]. This research incorporated Random Forest into behavioural pattern classification because each participant’s data samples were few. The behavioural pattern data sets’ sizes ranged from 1371 to 1971. Due to size limitations, the Random Forest classifier incorporated randomness in enhancing its accuracy and combatting the overfitting problem other classifiers face in the presence of low-sample datasets. Overfitting is not peculiar to Random Forest because the classifier uses the mean of all predictions from the decision trees and cancels out the biases. The classification report from the Random Forest algorithm is shown in Table 4.

**Figure 17:**
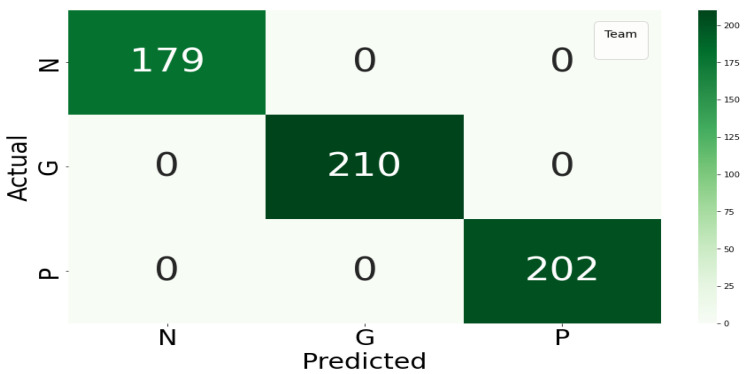
Confusion Matrix from Random Forest classifier.

From the classification report in Table 4, precision, recall, accuracy, macro mean, and weighted mean were used as metrics to evaluate the classifier. The accuracy for the classifier was 0.98. The precision for N and P classes was 1, indicating that the classifier was able to label patterns belonging to the classes appropriately. The precision for the G class was 0.95, which was lower than the precision for other classes but still acceptable. The recall for N and G was 1, indicating that the classifier was able to identify all true instances of these classes. The recall for the P class was 0.95, which is lower than the precision for the other classes, but this value is still acceptable. Classes G and P had the same F1 score of 0.97, which is lower than the F1 score for class N, which was 1. These F1 scores indicate that at least 97% of the positive predictions were correct. Furthermore, the micro and weighted means were the same, indicating that all patterns were correctly classified into appropriate classes, even in the presence of varying instances in each class label. The confusion matrix for the classifier is shown in Figure 17.

The confusion matrix in Figure 17 showed 11 instances where the Random Forest classifier misclassified the behavioural patterns with the actual P label into the G class. However, these instances are few when compared to the size of the dataset. For this participant, the size of the behavioural pattern dataset was 1970. With a train/test split ratio of 0.8, the training dataset was 1379, while the size of the predicted test set was 591, which is indicated as the sum of the support data in Figure 17. The performance of the classification is reflected in the classification report. The confusion matrix showed that the third evaluation property (behaviour pattern classification) in Table 1 was verified by showing that the classification component of the PBC model can classify identified behavioural patterns using existing classifiers. The summaries for the classified behavioural patterns are provided in Table 5.

Table 5 shows each participant’s total number of patterns in the dataset, the number of patterns classified as poor, good, or neutral, and the mean length of each participant’s patterns. From the length information, P5 had the highest mean pattern length and the lowest number of patterns, and P1 had the highest number of behavioural patterns. Behavioural pattern length did not determine the proportion of patterns in any category, as indicated by P5. Similarly, P3 had the smallest mean pattern length with a low number of good patterns. Concerning the content of the behavioural patterns, all the participants had more neutral behavioural patterns than other behavioural classes, with good patterns being the least. Therefore, the second question has been classified through the incorporation of existing classifiers for the classification of behavioural patterns. Therefore, RQ_4_ was answered through the incorporation of an existing Random Forest Classifier into a behavioural pattern classification task. The third evaluation property (behaviour classification) was achieved. The purpose of the classified behavioural patterns was to ensure that the relevant features required for change detection and analysis in Section 8.7 and Section 8.8 are captured. For change detection, Section 8.6 describes how the participants’ productivities were quantified. 

### 8.6. Productivity Estimation

The research used Rescue time to collect the activity dataset. As a self-monitoring tool, it had a quantification effect, which was on the side of the tool due to its automated nature, i.e., the programmer used specific information for productivity estimation. Therefore, the process of productivity estimation through quantification was based on what the programmer considers productive or not productive. The quantification from these tools was not fit for this research because some misclassifications were observed. New productivity estimation was based on the activity re-categorisation conducted in Section 7.3.2 and behavioural pattern classification in Section 8.5.

Based on behavioural pattern classification, the research conducted productivity estimation through quantification by generating the following new features.

Weight (W): weights were created for each behavioural class through the following mappings

- If a pattern had a P tag, a weight of 1 is assigned.
- If a pattern had an N tag, a weight of 2 is assigned.
- If a pattern had a G tag, a weight of 3 is assigned.

Productivity (*P*): Productivity values were generated through the following formula

$$P=\left(W*L\right)/\sum \left(W\right)$$

where *W* is the weight assigned to each behavioural class, *L* is the behavioural pattern length, and *P* is the productivity. The subsequent sections used these new features for time series analysis and change detection. The details of the time-series analysis and change detection are given in Section 8.7 and Section 8.8.

### 8.7. Timeseries Exploration and Analysis

Exploration through time series analysis was conducted because the digital activity dataset collected in this research had a timestamp as one of its features, making it a time series dataset. The data were collected at a granular interval of 1 min but have been aggregated to 30 min intervals during behavioural pattern extraction as discussed in Section 8.2.

**Figure 18:**
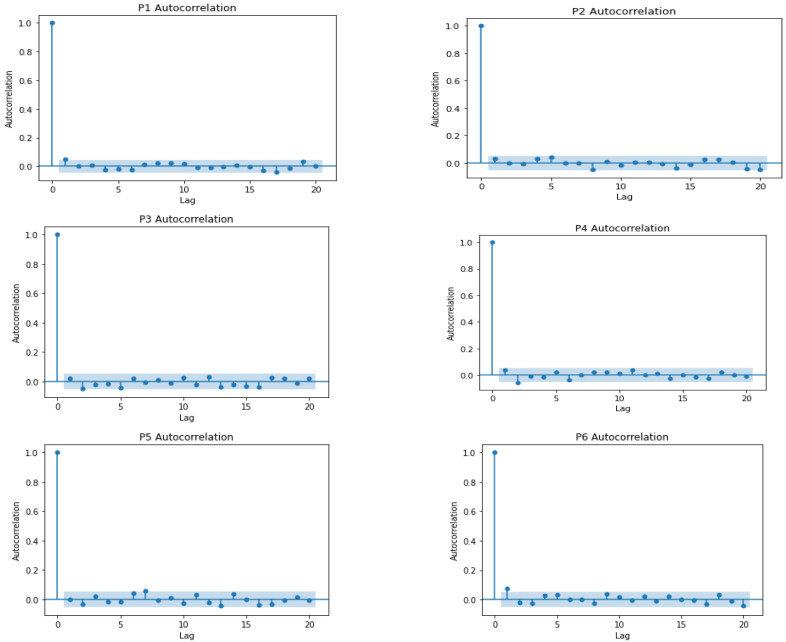
Autocorrelation results.

Autocorrelation is essential for any time series analysis because it ensures that the previous values in the time series do not affect or determine current or future values. The presence of autocorrection in time-series data affects sampling distribution [116] and helps to highlight potential problems before engaging in any thorough analysis and modelling. Autocorrelation in time series data is a pointer to repeating patterns, seasonality, and non-stationarity. Seasonality means variation of a specific frequency happening at a constant time interval. Seasonality in productivity is possible and can happen because of several factors highlighted in Section 2.6. Stationarity means that the time series has three properties: constant variance, constant mean, and covariance, dependent on the time interval between data points. A stationary time series is always desired for any time series because it is easy to analyse and can be modelled using lesser parameters. However, there may be a change, but it will always return to its constant mean, making it easier to analyse and model [117]. Seasonality and stationarity are always revealed during autocorrelation analysis because a highly correlated dataset implies non-stationarity and the presence of seasonality. Therefore, there must not be any autocorrelation in time series data. Figure 18 shows the autocorrection results for the participants’ behavioural data.

The blue region in Figure 18 represents the 95% confidence level and the region where the autocorrelation results are meaningful. The autocorrelation results show no significant autocorrelation for all the participants. The bars are within the 95% confidence level. Therefore, previous productivity values do not affect current or future productivity values. The absence of autocorrelation also implies that the behavioural datasets are stationary with no seasonality [118]. 

**Figure 19:**
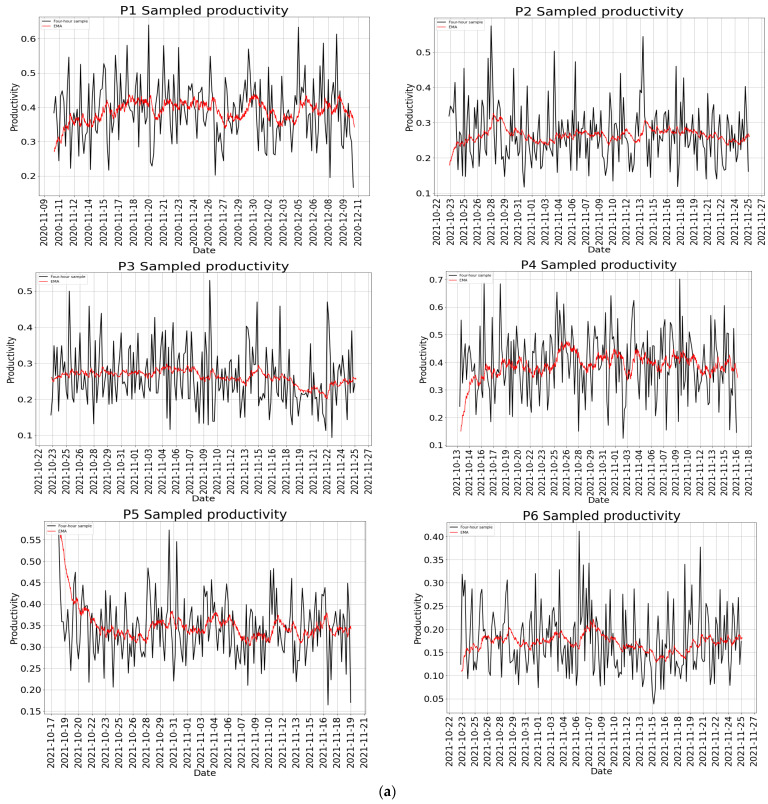

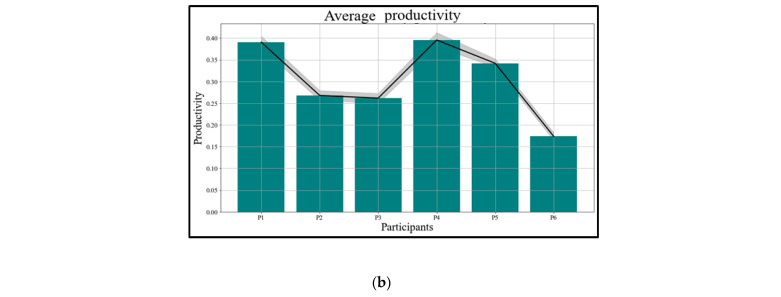
(**a**) Sampled productivities at 4 h frequency. (**b**) participants’ productivity summary.

The research conducted a time-series analysis on the productivity and timestamp columns. Workers’ productivities were visualised through line graphs. The productivity datasets were resampled at four hours to have a less dense view of the participants’ productivity over one month. The four-hour sampled datasets were used in Figure 19a,b to observe how productivities differ before and after lunch. Additionally, the Exponential Moving Average (EMA) graphs were imposed on the resampled charts to aid visual inspection of the trend. Figure 19a shows the EMAs for the participants.

Figure 19a shows the four-hourly sampled data and each participant’s productivity EMA. The EMA line graphs in red colour were included to aid trend detection through visual inspection because the sampled data are not visually suitable to identify trends. From visual inspection, all the participants had both upward and downward trends, signifying that the participants’ productivities were not constant. Still, these varying trends had no impact on the stationarity of the dataset, i.e., they do not affect the means of the distributions. A more insightful visualisation is the productivity summary for each participant for the one-month data collection period through the bar graphs. Figure 19b shows the bar graphs.

**Figure 20:**
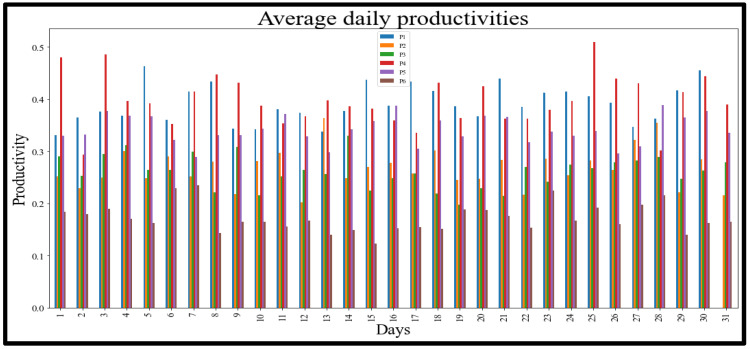
Daily Productivities.

Figure 19b shows all the participants’ average productivity for one month. From the bar graphs and the confidence interval line, the following can be inferred from the one-month data: P4 had the highest average productivity, followed by P1 and P5, P2 and P3 had similar average productivity, and P6 had the lowest average productivity. Therefore, the most productive worker is P4, while the least productive worker is P6. Figure 20 presents a more insightful exploration of workers’ daily productivity during the one-month data collection period.

**Figure 21:**
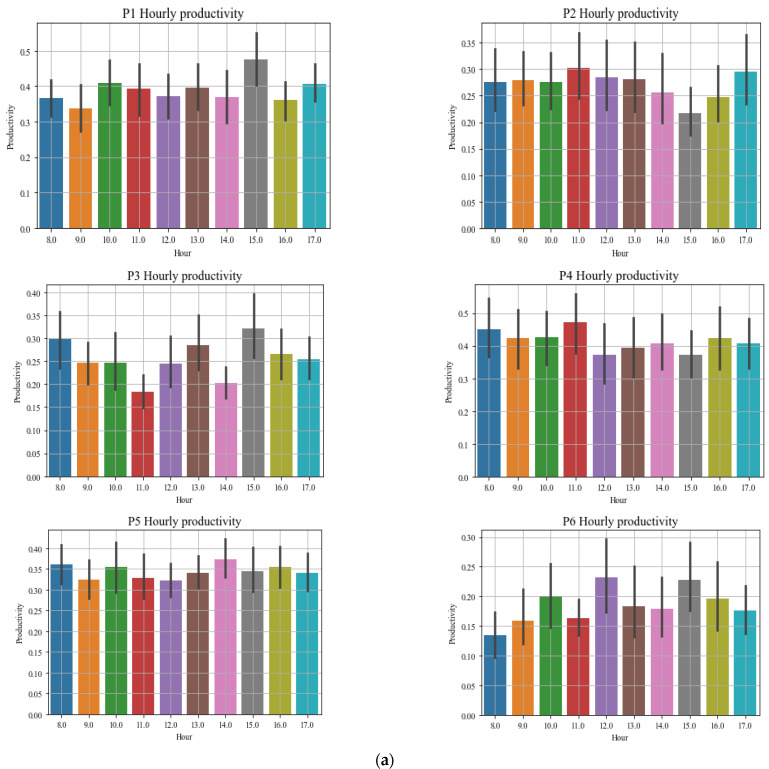

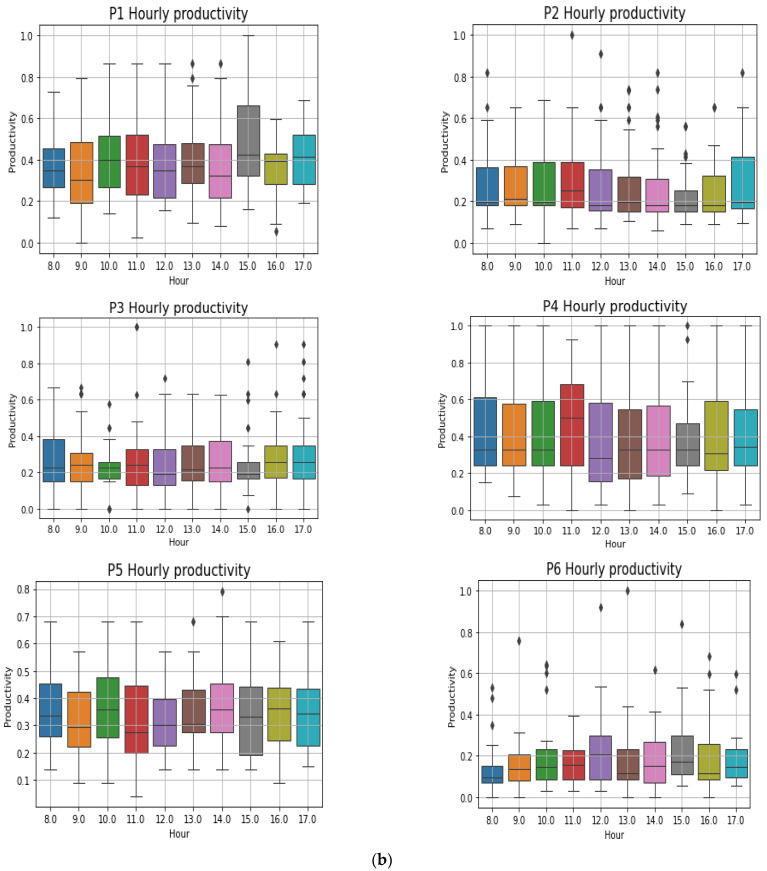
(**a**) Hourly productivities I. (**b**) Hourly variation in productivities.

Figure 20 shows the daily productivity of the participants. Similar to the previous observation in Figure 19b, the following was observed during the one-month data collection: P4 had the highest mean daily productivity, followed by P1 and P5. P6 had the lowest mean daily productivity. P2 and P3 had similar daily mean productivity. Productivities varied for all the participants per day. Finally, Figure 21a,b represent workers’ hourly productivity graphs with bar and box plots.

Figure 21a shows that participants had different peak productive hours, which can be before or after noon or at noon. The following was observed in Figure 21a, P1 was most productive at 15 h, after lunchtime, P2 and P4 were most productive at 11 h, before lunchtime, P3 was most productive at 17 h, after lunch, towards closing time, P5 was most productive at 14 h, shortly after lunchtime, P6 is most productive at 12 and 15 h, before or after lunchtime.

From Figure 21a, the participants’ peak productive moment before or after lunch did not signify the importance or effect of lunch on productivity because lunch’s impact on productivity was not within the scope of this research. Future studies can study the association between lunch/lunchtime and productivity. Figure 21b represents hourly productivity variation. 

Figure 21b shows that each participant’s productivity varies across hours of the day because each hourly productivity is different from other hours. It was observed that P1 had significant variations among each hour and had more stable productivity than other participants. P2 had no variation between 8 and 9 h, 10 h, 11 h, 13 h, and 14 h. P2 had moderate hourly variations when compared with other participants. P3 had no variation between 11 and 12 h, 10 and 15 h, 16 h, and 17 h. P3 had the lowest and moderate variations. P4 had the most considerable hourly variation when compared with other participants. P5 had significant variations but was stable in the afternoons. P6 had little hourly variations, increasing in the morning and decreasing in the afternoons. P2, P3, and P6 had fewer variations than P1, P4, and P5. 

The hourly variations implied that the participants’ work engagement and productivities were not the same. Several individual factors may be responsible for these variations, which were not reflected in the data collection process and were not within the scope of this study.

### 8.8. Change Detection

Isolation Forest is a collective approach used to identify changes/anomalies. It starts with a random feature and selects a partition between the highest and the lowest values to divide a sample. This process continues until all the samples are divided. The Isolation Forest is constructed by including several isolation trees divided into several features. The number of divisions required to separate a sample is the path length from a root to a leaf [67]. 

Isolation Forest was established on Extra Trees [68], in which each separation is random. A sample close to the root (i.e., short path length) can be distinguished effortlessly and is easier to separate from samples more comparable to the leaves. Additionally, change/anomalies will have a smaller mean path length than normal samples. When there is a sample at the leaf, in the deeper part of the tree, the score will be close to 0. A shallow sample close to the root will have a high score of close to 1 [69].

The selection of isolation forests for detecting a change in productivity was motivated by its ability to detect a conservable number of changes and by the work of Shiotani and Yamaguchi [70]. The authors demonstrated the power of Isolation Forest to detect a change in patients’ physical condition (heart rate and dietary intake) in a care facility with smart devices. Furthermore, Isolation Forest works well for low- and high-dimensional data, even in the presence of unimportant attributes and in contexts with no anomalies or where change is absent [67]. 

The approach followed by Chen et al. [119] and Tao et al. [120] was incorporated. Chen et al. [119] evaluated the Isolation Forest algorithm’s performance by comparing it with other unsupervised change detectors. Tao et al. [120] evaluated Isolation Forest by estimating the accuracy as the proportion of instances in the dataset that was correctly detected. This study initially evaluated the algorithm by fitting the first participant’s productivity dataset with the Isolation Forest using Chen’s approach [119]. The accuracy scores for the normal and change values were obtained as an unsupervised learning algorithm. The accuracy score for the normal class was obtained by finding the ratio of correctly predicted normal values to the total number of normal values in the dataset. 

**Figure 22:**
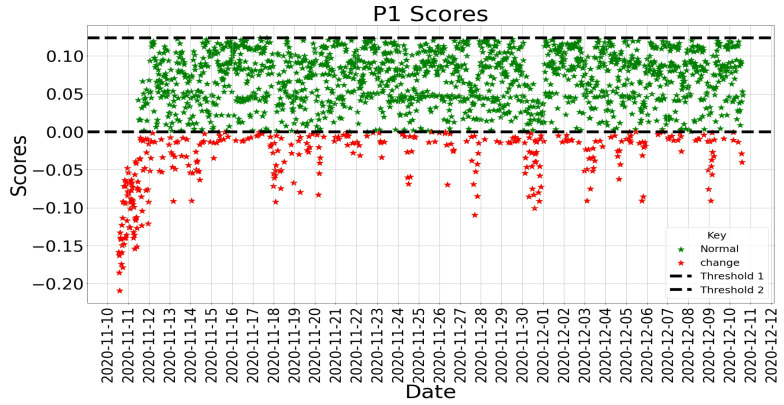
Scores from Decision Function.

A similar approach was applied to the change class. The results showed an accuracy of 0.86 for normal and 0.71 for change values. After the initial evaluation, changes in the other participants’ productivity were detected. The scores were retrieved from the algorithm’s decision function, as shown in Figure 22.

Figure 22 shows the regions for the normal and change scores for P1. The green and red regions represent normal and change scores, respectively. Figure 16 shows that productivities with low change were assigned negative scores, and normal productivities were assigned positive scores, confirming that change values will have a smaller path length than normal values [69]. With change detection in behavioural data sets, the fourth evaluation property (change detection), specified in Table 1, was achieved. Therefore, the third question has been answered through the incorporation of existing change detection algorithms into the productivity datasets.

**Figure 23:**
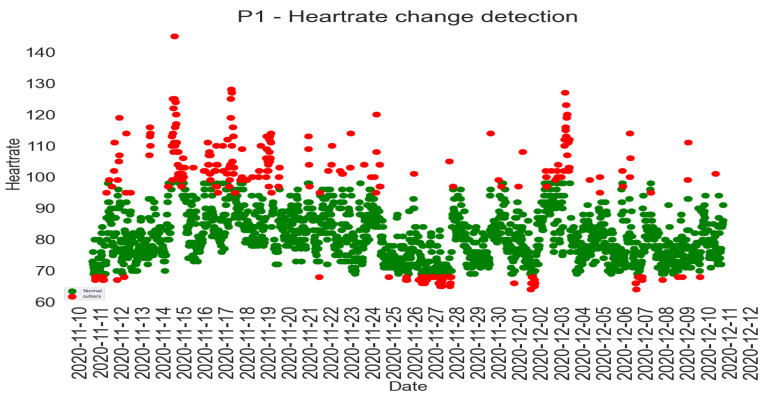
Heart rate Change detection.

Change detection using the Isolation Forest was performed on the heart rate data set to see whether there are change points and whether these change points are related to the productivities. The results for P1 are shown in Figure 23.

**Figure 24:**
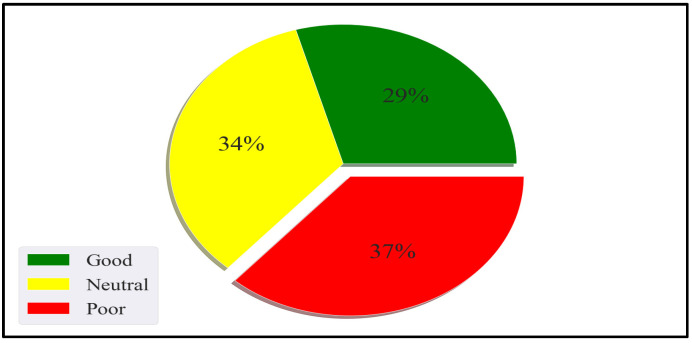
Heart rate class distribution (*n* = 6).

Figure 23 shows how the Isolation Forest algorithm demarcated the change points from the normal heart rate points. The green region represents the participants’ normal heart rate values, while the red region represents the participants’ heart rate-change points. The plot shows that there are change values that are above and below the normal values. However, the focus of this study was on the values that were above the normal heart rate points for each participant because, medically, a high heart rate could signal potential health problems [121]. Pie charts were used to obtain an overview of the distribution of behavioural classes in the upper change points. The summary pie chart is represented in Figure 24. 

Figure 24 illustrates all the participants’ class distribution summaries. According to DiGiacinto and Seladi-Schulman [35], the mean heart rate value for adults ranges from 60 bpm to 100 bpm. In this study, high heart rate values were not interpreted as an indication of poor behaviour if these upper heart rates change values were within the range of 60 bpm to 100 bpm. This study examined the proportion of good, poor, and neutral behavioural patterns associated with the combined upper change heart rate dataset. Thirty-seven percent of the upper change heart rates, which was the most significant proportion, was for poor behavioural patterns; twenty-nine percent of the upper change heart rate, which was the minor proportion, was for good behavioural patterns; thirty-four percent of the upper change heart rates were for the good behavioural patterns. The combination of yellow and red portions (total = seventy-one percent) of the total distribution implied that most participants’ heart rates did not map to good behavioural patterns. However, proportional analysis through pie charts is insufficient to analyse the association between high heart rate values and behavioural pattern classes/productivity. Correlation analysis was incorporated to reveal the association between these variables, which is depicted in Table 5.

Table 6 shows that for all the participants, there were weak negative correlations between the upper heart rate change values and productivity values for P2, P3, P5, and P6, and weak positive correlations were observed for P1 and P4. However, since these correlation values are close to zero, it can be inferred that the changes in participants’ productivity are not associated with changes in heart rate and that high heart rate values are not associated with high or low productivity.

### 8.9. Change Analysis

**Figure 25:**
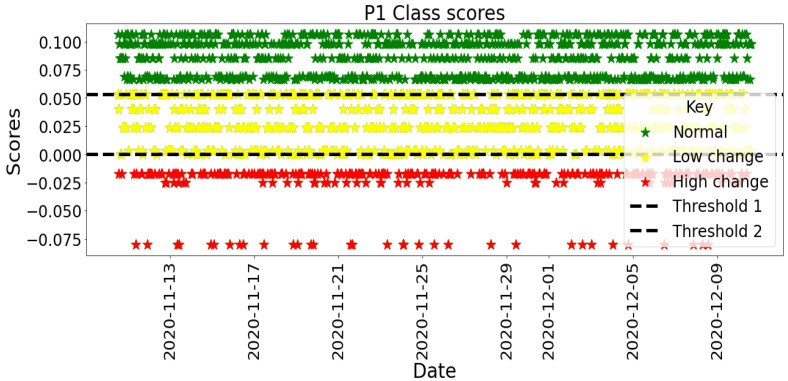
Further Change Division for P1.

For change analysis, the productivity changes were divided into low and high changes, respectively. Further change division was based on the work of Elbayoudi [122], where the authors divided an individual’s occupancy behaviour into three: normal, warning, and abnormal levels. In this study, this division determined the extent of a worker’s productivity change and the kind of intervention to provide. Some changes in productivity values may not warrant serious attention, and these can still be managed. A typical change division for a participant is shown in Figure 25.

With the scores derived from the Isolation Forest’s decision function, as depicted in Figure 25, the change points were represented outside the normal productivity region. The graph was derived by plotting all the scores in green lines and overlaying low and high changes. The green region represents a region for normal productivity scores. The yellow region represents low changes in productivity, while the red region represents significant changes in productivity. 

The rationale for creating new change classes is to help determine the severity of a change in workers’ productivity and to recommend appropriate interventions. The green-only region represents the worker’s normal productivity, where there is no change, and is the recommended productivity value for optimal work performance. The yellow region represents a region of workers’ productivity with little change. Productivity values in this region do not call for serious concern, but workers need to be aware of these little changes to avoid further change. The red region signifies a region of workers’ productivity with significant changes. Productivity values in this region call for immediate concern and action towards improving the worker’s productivity. Based on change division, the study recommends the provision of change-specific interventions. Therefore, the intervention for the low-change region will differ from the intervention for the high-change region. Similarly, there will be an intervention for the no-change region. 

The research has demonstrated the abilities of the user model, behaviour model, and classification components of the PBC Model. The intervention component will be communicated via future publication.

## 9. Discussion

The study focused on the design and evaluation of the PBC model. The PBC model was designed by using the DSR methodology as a guideline for model development and evaluation. Therefore, the development of the PBC model answered RQ_1_ by emphasising the inclusion of smart environment, user model, behaviour model, classification, and intervention as the core components that are needed in modelling positive behaviours in smart environments. The study evaluated the PBC model through user modelling, behavioural pattern identification, classification, and change detection. However, before these activities, exploratory data analysis was performed on the dataset. Heart rate data exploration revealed that the participants had higher heart rates than the specified values for their age but were still within the acceptable range of normal heart rate values. Furthermore, work tab engagement revealed that the participants engaged with Utilities, Message, Social, Portal, Entertainment, Social, and Business because these categories had the highest frequency in each participant’s digital work data. 

User modelling was undertaken by extracting the activity models from event logs using a Heuristic miner. The extracted activity models reveal the weighty paths, which signify the frequent transitions from one activity to the other. Behavioural pattern identification was made by extracting user models from the activity datasets of each participant. This study incorporated the extracted activity models and internal context as specific outputs from user modelling. The user (activity) models were extracted using a Heuristic miner. Using the evaluation technique by Rojo et al. [111], the study achieved a result that was similar to the result achieved by [111], where the authors extracted core social workflows from activities of daily living data using a specified weight. In this study, all relevant activities were retrieved with a weight of 0.4. The user modelling conducted in this study was not evaluated because of the use of a Heuristic miner from process mining. Process mining algorithms are usually evaluated through conformance analysis, which is beyond the scope of the study. However, other approaches to user modelling can be explored from the machine learning domain. The extraction of activity models, as a specific output of user modelling from the digital work dataset, answered RQ_2_. The study showed that a user model could provide specific information about various aspects of users by revealing activities and associated heart rates.

The activity models were used to identify participants’ behaviours through pattern identification and extraction. The behavioural pattern extraction performed in this study was based on the CM-SPADE algorithm, a depth-first strategy with a vertical database structure for faster processing. Existing sequential pattern mining algorithm evaluations are based on performance comparison. However, this study incorporated only the CM-Spade algorithm in extracting behavioural patterns. Huynh er al [41] and Fournier-Viger et al. [46] evaluated the CM-SPADE algorithm through computation time and memory usage. However, the focus of the behavioural pattern extraction conducted in this study was not to ascertain how well it outperformed other algorithms in terms since a particular type of dataset and CM-SPADE were used. The study recommends future studies on how pattern recognition can be evaluated without these dimensions, especially when only one algorithm has been incorporated. N-grams and Markov chains were used to graphically view the top behavioural patterns based on each participant’s mean pattern length. Similar to the findings from Amato et al. [113], the Markov chains provided a better view of the behaviour transitions through probability computation. The findings from behaviour modelling provided a new approach to behavioural modelling, which is human behaviours could be modelled from user models using existing pattern recognition algorithms. The user models in this study allowed the discovery of core activities in which behaviours were based on. 

Behavioural pattern classification provided detailed insights into individual-specific work engagements based on the proportion of each behavioural class, as summarised in Table 5. The Random Forest classifier had an accuracy of 0.98 and performed better than the results obtained from the multi-class classification in [52], where an accuracy of 0.80 was obtained during the multi-class classification of an IoT dataset. Behavioural pattern classification paved the way for behaviour (productivity) quantification, an important step for behaviour change identification. 

With weighted productivity, time series exploration and analysis revealed that the participants’ productivities were not autocorrelated, i.e., no association exists between previous and present productivity values. Therefore, an individual can be productive at one moment and less productive at another moment. All the participants’ productivity data had both upward and downward trends. The participants’ productivities are not constant, but these varying trends had no impact on the stationarity of the dataset. They do not affect the distributions’ means; all the participants had different mean productivities, with P4 being the most productive and P6 being the least productive worker for the data collection period. All the participants had different mean daily productivity, with P4 having the highest mean daily productivity and P6 having the least mean daily productivity during the data collection period. All the participants had different peak productive hours, with no association with lunchtime. However, the reasons for these differences cannot be ascertained because it is not within the scope of the current research.

Change detection was performed using the Isolation Forest algorithm. The findings from the Isolation Forest algorithm application revealed two issues: (i) Participants working in offices had changing productivities at some point in their workday. Therefore, productivities can change based on some factors identified in Section 2.6. (ii) The identified changes had negative scores assigned to them, while normal productivities had positive scores assigned to them. The change points had smaller path lengths than normal points. The result is similar to the work of Tao et al. [120], where detected anomalies had negative scores. Furthermore, the results from change detection showed an accuracy of 0.86, which is slightly lower than the work of Tao [120], where an accuracy of 0.87 was recorded. The study proposed the use of change division in developing and delivering interventions to people. Change analysis showed that change points could be further divided depending on the severity of the observed change. The division will help design and deliver appropriate interventions based on the observed change. The intervention can be in terms of simple warnings or the proposition of specific actions to improve behaviour. However, the change-based intervention will be investigated in a future study. The evaluation of Isolation Forest’s performance in this study was similar to the result from [120], indicating a consistent performance across different datasets. 

The Isolation Forest algorithm was applied to the participants’ heart rate datasets. Two results were revealed from this step. Internal context modelling can be conducted through the heart rate dataset by creating profiles for normal and changing heart rate values. The upper change heart rate data had neutral and poor behavioural patterns, but correlation analysis revealed weak associations between the high heart rate values and productivity. The findings revealed that for this study, an increase in heart rate did not lead to a corresponding increase or decrease in the participants’ productivity.

The PBC model was evaluated in parts, i.e., the components of the PBC model were evaluated for the properties specified in Table 1. Therefore, RQ_5_ was answered through the effectiveness of the activity model in representing the core activities engaged by the participant, the extraction of the behaviour model from the user model, the classification of the behaviour model, and the quantification of behaviour for change-based intervention. The study provided a new model for modelling behaviours in smart environments. Another novel idea from the study is the modelling of behaviours with inputs from the user model. 

## 10. Contribution

The contribution of this study is in two parts: theoretical and practical contributions. Theoretical contribution highlights how the body of knowledge has been extended through the study, while practical contribution highlights how a solution was provided for an identified problem. These contributions are discussed in subsequent sections. 

### 10.1. Theoretical Contribution

The theoretical contribution of this research is in three parts: 

The first is the identification of behavioural problems in office environments. Behavioural problem identification was achieved through the incorporation of the relevance cycle of the DSR methodology. Behavioural problems identified in offices were resource use, multitasking, cognitive, sedentary behaviour, and social media use behavioural problems. The relevancy cycle identifies problems, requirements, and opportunities for solving problems in a particular domain.

The development of the PBC model through the rigour of the DSR methodology, which focuses on identifying existing literature, theories, models, and constructs, identifying their limitations, and developing an artefact, can extend the knowledge base of the problem domain. The identified problems were considered during the PBC model’s design. The model objectives were drafted as solution objectives by identifying and analysing the current limitations of the existing behavioural models and solving them by including them in the proposed model.

Therefore, the development of the PBC model represents a significant theoretical contribution to the study. The PBC model can be used by researchers to model and provide solutions to behavioural problems in all the use cases specified in Section 6.

### 10.2. Practical Contributions

Practically, the study contributed by demonstrating the effectiveness of the PBC model through a naturalistic-summative evaluation. The study focused on the PBC model’s presentation, instantiation, and evaluation through data collection, user modelling, behavioural pattern identification, classification, and change detection. Therefore, the practical contributions of the study are behavioural modelling through pattern identification from user models (activity model). This study incorporated activity models and heart rate as an internal context. These variables were used as specific outputs from user modelling. Using Heuristic miner, the user (activity) models were extracted from digital activity datasets. Using the evaluation technique by Rojo et al. [111], the study achieved a result that was similar to the approach used by Rojo et al. [111], whereby relevant activities were obtained at a particular arc weight. Therefore, the first practical contribution of the study is the extraction of the activity model as specific incorporation of user models. The study showed that a user model could provide specific information about various aspects of users by revealing activities and associated heart rates.

The second practical contribution of this study is the creation of behavioural models from activity models through behavioural pattern mining. The behavioural pattern extraction performed in this study was based on the CM-SPADE algorithm, a depth-first strategy with a vertical database structure for faster processing. Existing sequential pattern mining algorithm evaluation used performance comparison with other algorithms. However, this study incorporated only the CM-Spade algorithm in extracting behavioural patterns. The focus was not to ascertain how well it outperformed other algorithms. Therefore, N-grams and Markov chains were used to graphically view the top behavioural patterns based on each participant’s mean pattern length. The third practical contribution from this study is behavioural pattern classification, which provided detailed insights into individual-specific work engagements based on the proportion of each behavioural class, as summarised in Table 5. The study used existing evaluation metrics to evaluate the classification performance of the Random Forest classifier. The classification report and confusion matrix from the Random Forest classifier demonstrated that the behavioural patterns were well classified into appropriate classes. For the classification carried out in this study, the Random Forest algorithm performed better than the results obtained from the multi-class classification conducted by Khadse [52]. The fourth practical contribution is behaviour (productivity) estimation, meaning that human behaviour can be quantified for further analysis. Using the quantified productivity, time series exploration and analysis revealed that the participants’ productivities were not autocorrelated, i.e., no association exists between the previous and present productivity values.

The fifth practical contribution is change detection through the Isolation Forest algorithm. Change detection provided further insights and understanding of participants’ work behaviours at specific times. The sixth practical contribution is the motivation for intervention provision and delivery through change division. The change division in the study revealed that some poor behaviours might not have critical effects. However, people need to be aware of the small deterioration caused by these poor behaviours to avoid further deterioration. The seventh practical contribution is the determination of change points in heart rate values and their associated effects on behaviour and productivity.

## 11. Conclusions

The study aimed to present, instantiate, and evaluate the PBC model. The PBC model was developed through consultation with the existing theories and models, as stipulated by the rigour cycle of the DSR methodology. Instantiation was achieved by setting up personal smart environments in workers’ offices for digital activity and heart rate data collection. The merged dataset was used to evaluate the core components of the PBC model.

The essential features for behavioural modelling were generated from the merged dataset, which allowed the generation of user models, thereby achieving the first evaluation objective of user modelling and verifying the first solution objectives. Behaviour pattern extraction was carried out as the core activity of behavioural modelling and was achieved through the CM-SPADE algorithm. The ability of the behavioural model component to adequately extract behavioural patterns from a user model served as a verification of the second evaluation property (behaviour pattern recognition). A significant component of the PBC model is behaviour classification, which was achieved through a string function and the Random Forest classifier. The performance of the behaviour classification module, as reflected in the classification report and confusion matrix, verified the third evaluation property.

The study evaluated the PBC model using office environments as the domain. Workers’ productivities were estimated. However, productivity estimation does not provide insight if changes in productivity are not identified. Time series analysis was conducted on the productivity dataset, thereby paving the way for change detection through the Isolation Forest algorithm. The ability of the PBC model to detect change confirmed the fourth evaluation property. The change class was divided into two, low and high change, to enable proper intervention provision because some changes may not elicit serious concern. Change analysis was carried out using the new division, and it summed up the evaluation goal of the study. 

The current evaluation conducted in this research is limited to an office environment. The research is limited by the small number of participants (*n* = 6), with a personal smart environment set up in each worker’s office. More research with a larger number of participants is suggested for future work. Additionally, the performance of the PBC Model needs to be ascertained in smart environments with multiple users in a single space. In modelling user behaviour, the authors will study behavioural problems at the unit level by splitting problems into smaller problems, providing solutions to these smaller problems, and combing the problems to generate the final solution through the divide and conquer techniques. This manner will allow easy navigation through the components of the PBC Model. 

The PBC model is not limited to the techniques specified with each component. Therefore, the authors shall conduct more experiments with relevant verifications and advanced statistical and machine learning techniques using the domains specified in Section 6. future users of the PBC Model are also encouraged to incorporate advanced techniques while modelling user behaviour. At the moment, the user model, behaviour model, and classification components have been evaluated. The research will be furthered by developing, delivering, and evaluating change-based interventions for the PBC Model. The outcome of the interventions will be communicated in a future study.

## Figures and Tables

**Table 1:** Evaluation Process.

Step	Details	Mapping
1.	Evaluation goals	Rigorous evaluation helps ensure that artefact works in a realistic environment.
2.	Select suitable evaluation technique(s)	The human risk and effectiveness strategy was used to evaluate the PBC model’s behavioural model and classification components.
3.	Decide on the properties to evaluate	User model identification, behavioural pattern identification, behavioural pattern classification, and change detection.
4.	Plan each evaluation episode	Naturalistic summative evaluation.

**Table 2:** Heartrate Summary (*n* = 6).

Participant	Measure	Value (bpm)
P1	Mean	82.11
	Median	80.00
	Standard deviation	11.26
P2	Mean	66.52
	Median	65.00
	Standard deviation	10.75
P3	Mean	79.31
	Median	78.00
	Standard deviation	19.92
P4	Mean	89.00
	Median	81.00
	Standard deviation	9.85
P5	Mean	81.82
	Median	81.00
	Standard deviation	10.28
P6	Mean	88.29
	Median	88.00
	Standard deviation	7.79

**Table 3:** Behavioural patterns summary (*n* = 6).

P#	Event Log Size (n_1_)	No of Behavioural Patterns (n_2_)	Mean Length
P1	8691	2491	5.21
P2	6335	2691	5.44
P3	4913	1394	3.52
P4	16,766	1417	4.49
P5	2450	1371	5.90
P6	10,260	1442	4.21

**Table 4:** Classification report.

	Precision	Recall	F1-Score	Support
N	1.00	1.00	1.00	179
G	0.95	1.00	0.97	210
P	1.00	0.95	0.97	202
Accuracy			0.98	591
Macro avg	0.98	0.98	0.98	591
Weighted avg	0.98	0.98	0.98	591

**Table 5:** Summaries for the classified patterns for all participants (*n* = 6).

P	Total No of Patterns	No of Poor Patterns	No of Neutral Patterns	No of Good Patterns	Mean Pattern Length
P1	1970	650	700	620	5.21
P2	1421	472	501	448	5.44
P3	1390	450	520	420	3.52
P4	1416	474	514	428	4.49
P5	1370	450	500	420	5.90
P6	1441	464	512	465	4.21

**Table 6:** Correlation summary (−1 ≤ r ≤ 1).

Participant	r
P1	0.04
P2	−0.04
P3	−0.02
P4	0.03
P5	−0.04
P6	−0.05

## Data Availability

ADW Datasets–OneDrive (sharepoint.com), accessed date 21 December 2021.
